# Biologically Active Compounds of Plants of the *Atraphaxis* Genus: Chemical Composition and Immunomodulatory Evaluation

**DOI:** 10.3390/ijms262110301

**Published:** 2025-10-23

**Authors:** Meruyert D. Dauletova, Almagul K. Umbetova, Nazym S. Yelibayeva, Gauhar Sh. Burasheva, Aisulu Zh. Kabdraisova, Zhanat Zh. Karzhaubekova, Yuliya A. Litvinenko, Zhanibek S. Assylkhanov, Dmitriy Yu. Korul’kin

**Affiliations:** 1 Department of Chemistry and Technology of Organic Substances, Chemistry of Natural Compounds and Polymers, Al-Farabi Kazakh National University, Al-Farabi Ave. 71, Almaty 050040, Kazakhstan; dmd_09@inbox.ru (M.D.D.); nazym_yelibaeva@mail.ru (N.S.Y.); gauharbur@mail.ru (G.S.B.); yuliya_litvinenk@mail.ru (Y.A.L.); zhanik1903@list.ru (Z.S.A.); dmitriy.korulkin@kaznu.edu.kz (D.Y.K.); 2 Laboratory of Pharmaceutical Chemistry and Pharmaceutical Technology, Scientific Center for Anti-Infective Drugs, Al-Farabi Ave. 75B, Almaty 050060, Kazakhstan; zhaksais@gmail.com; 3 Institute of Botany and Phytointroduction, Forestry and Wildlife Committee, Ministry of Ecology and Natural Resources, Almaty 010000, Kazakhstan; kalidium2012@gmail.com

**Keywords:** *Atraphaxis virgata*, bioactive compounds, flavonoid glycosides, phenolic acids, supercritical CO_2_ extraction, GC–MS, antioxidant activity, immunostimulation

## Abstract

This study systematically investigated lipophilic and polar metabolites of *Atraphaxis virgata* (Polygonaceae) and assessed its immunomodulatory activity in vivo. Supercritical CO_2_ extraction of the aerial parts yielded a lipophilic fraction analyzed by means of gas chromatography–mass spectrometry (GC–MS), which identified 42 compounds, including fatty acid esters, sterols, hydrocarbons, and terpenoids. The residual plant meal was subjected to ultrasound-assisted extraction with 70% aqueous ethanol at 30–35 °C, using a solid-to-solvent ratio of 1:8 for 120 min. This polar extract was evaluated for amino acids, proteins, and carbohydrates, while solvent–solvent partitioning with chloroform, ethyl acetate, and water enabled isolation of phenolic- and flavonoid-enriched fractions. Six phenolic constituents, including four flavonol glycosides and two phenolic acids, were structurally confirmed. The extracts were rich in unsaturated fatty acids and water-soluble antioxidants, supporting their nutritional and pharmacological relevance. In vivo evaluation using a cyclophosphamide-induced myelosuppression model in Wistar rats demonstrated stimulation of erythropoiesis and leukopoiesis, confirming immunomodulatory potential. Collectively, this work provides the first comprehensive chemical and biological characterization of *A. virgata* and establishes a foundation for mechanistic studies and pharmacological validation.

## 1. Introduction

The genus *Atraphaxis* (*Polygonaceae*) comprises approximately 30 species distributed across arid and semi-arid regions, particularly in Central Asia, the Mediterranean, Eastern Europe, and China [1]. These plants thrive in dry, rocky terrains, foothills, and desert margins, demonstrating remarkable ecological adaptability [2]. Several species within this genus are known for their medicinal and economic importance, serving as traditional remedies and sources of bioactive compounds [3]. Additionally, *Atraphaxis* species contribute to ecological stability and function as forage for livestock [4]. Despite their potential, the chemical composition and pharmacological applications of many *Atraphaxis* species remain largely unexplored.

Phytochemical investigations of plants from the *Atraphaxis* genus have revealed a rich presence of secondary metabolites, including polyphenols, triterpenoids, alkaloids, essential oils, and lignans [5]. These compounds are associated with a wide range of pharmacological properties, such as antioxidant, anti-inflammatory, cytotoxic, and antimicrobial activities [6]. The identification of bioactive compounds is crucial for the development of new pharmaceutical agents, especially considering the increasing global interest in natural products for therapeutic use. Previous studies have demonstrated that *Atraphaxis* species contain valuable phytochemicals. Research has identified a range of polyphenolic compounds, such as flavonoids and tannins, which exhibit strong antioxidant properties [3]. Triterpenoids found in some *Atraphaxis* species have shown anti-inflammatory and cytotoxic effects [1]. Essential oils extracted from these plants contain volatile compounds used in traditional medicine and the food industry [7]. Phenolic acids, including caffeic and ferulic acids, have been reported in *Atraphaxis* species, highlighting their potential role in oxidative stress regulation [1]. Furthermore, certain species have been found to contain saponins and lignans, which are studied for their immune-modulating and anticancer properties [8]. Despite these findings, comprehensive phytochemical profiling of *Atraphaxis* species, particularly *A. virgata*, remains limited. Limited research has been conducted on the complete secondary metabolite composition of this species, making it a valuable subject for further exploration. *Atraphaxis virgata*, a little-studied species native to Kazakhstan, represents a promising candidate for in-depth phytochemical investigation. Previous reports on its medicinal use are scarce, and its full metabolomic profile remains unresolved. Given the expanding global interest in natural product-based therapeutics and the untapped biosynthetic potential of underexplored taxa, the chemical study of *A. virgata* is both timely and warranted. While prior research has provided initial insights into its flavonoid content, a broader investigation of its metabolite classes and pharmacologically relevant compounds is still lacking [9].

In addition to their antioxidant properties, flavonoids and phenolic acids are well known for their immunomodulatory effects. These compounds have been reported to influence cytokine production, regulate inflammatory mediators, and interact with signaling pathways such as NF-κB, thereby contributing to immune homeostasis [10,11]. Such evidence highlights the potential relevance of evaluating the immunomodulatory activity of *A. virgata* extracts alongside their phytochemical characterization.

To achieve a comprehensive phytochemical profile, we employed two complementary extraction approaches: CO_2_ extraction for lipophilic constituents (e.g., fatty acids, sterols, and hydrocarbons) and ethanol–water extraction of the plant meal for polar constituents (e.g., amino acids, flavonoids, and phenolic acids). The rationale was not to compare methods but to maximize coverage of secondary metabolite classes. This dual-extraction design was chosen to ensure that both lipophilic and polar metabolite classes were adequately represented, as reliance on a single method would bias the chemical profile toward only one polarity range.

Previous reports have described certain aspects of the amino acid and fatty acid composition of *A. virgata* [12]. However, these studies were limited in scope, and the present work provides a more comprehensive chemical and biological characterization, including novel identification of flavonoid glycosides, phenolic acids, and in vivo immunomodulatory activity.

The objective of this study was to establish a systematic workflow for both chemical and biological investigation of *A. virgata*, a wild species native to Kazakhstan, by employing advanced analytical techniques. Specifically, we aimed to characterize the lipophilic constituents of the CO_2_ extract by means of GC-MS, quantify amino acids, phenolic acids, flavonoids, proteins, and carbohydrates in the ethanol–water extract of the plant meal, and evaluate the immunomodulatory activity of the hydroethanolic extract in a cyclophosphamide-induced myelosuppression model in rats. Together, these objectives integrate phytochemical profiling with biological testing, providing both compositional and functional insights into this underexplored species.

## 2. Results and Discussion

### 2.1. GC–MS Characterization of the CO_2_ Extract of Atraphaxis virgata (Lipophilic Constituents)

**Figure 1:**
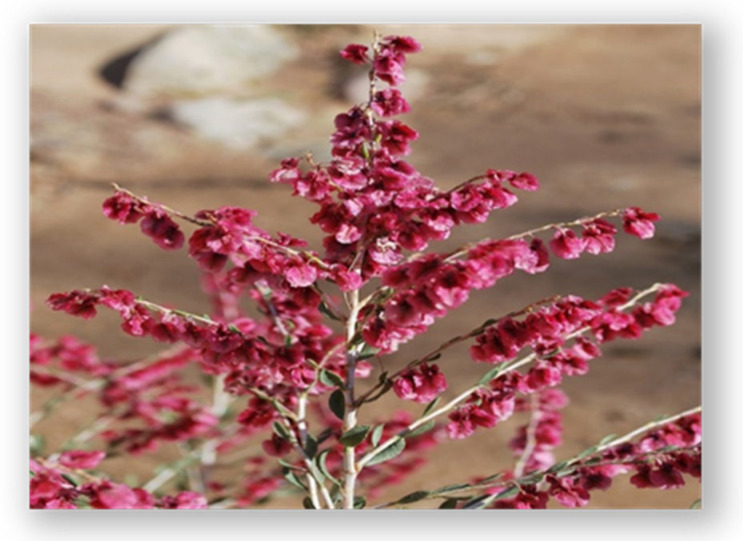
*Atraphaxis virgata* at the fruiting stage.

This research represents the first systematic investigation of both lipophilic and polar secondary metabolites of *A. virgata*, combining GC-MS analyses of the CO_2_ extract with complementary evaluations of the ethanol–water extract. Previous studies on the genus have been fragmentary, and our findings provide the most complete chemical and biological profile reported to date. Highlighting this novelty underscores the scientific value and originality of our work. Earlier reports on the genus were limited, and our study substantially extends the chemical and biological knowledge of this plant. In line with the experimental design, the CO_2_ extract was characterized by means of GC-MS for lipophilic constituents, while polar metabolites were analyzed only in the ethanol–water extract and its fractions. Dried aerial parts (stems, leaves, and flowers, Figure 1) of *Atraphaxis virgata* underwent CO_2_ extraction, yielding lipophilic compounds including fatty acids, mono-, di-, and triglycerides, phospholipids, sterols, sterol esters, glycolipids, and fat-soluble vitamins. These compounds constitute a structurally diverse group of bioactive molecules. Fatty acid esters, long-chain hydrocarbons, phytosterols, and terpenoids are well known for their antioxidant, anti-inflammatory, and lipid-modulating properties [10,11]. GC-MS analysis of the lipophilic fraction identified 42 fatty acid derivatives (Table 1). The major componets were ethyl linoleate (9,12-octadecadienoic acid ethyl ester) (28)—10.66%, nonacosane (39)—7.99%, palmitic acid (20)—7.67%, 2-nonadecanone (30)—7.40%, heptacosane (34)—7.17%, 2-nonadecanone (26)—5.14%, squalene (40)—4.73%, octacosanol (32)—4.44%, and ethyl hexadecanoate (21)—4.35%. Additional compounds included hentriacontane (41)—2.89%, ethyl 9,12,15-octadecatrienoate (29)—2.18%, and various minor substances [13,14].

As expected, CO_2_ extraction yielded primarily lipophilic compounds, whereas the ethanol–water extract of the plant meal provided access to polar constituents described in subsequent sections.

**Figure 2:**
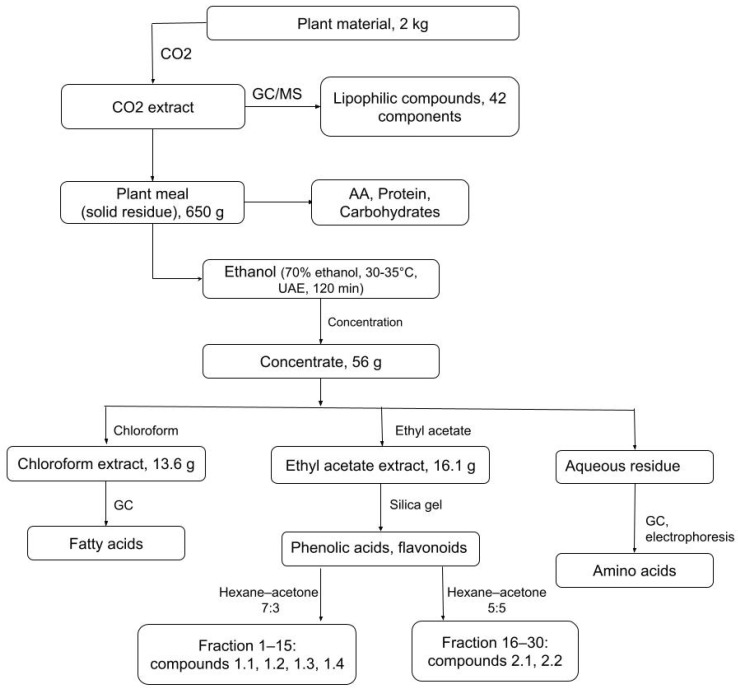
Flowchart of the sequential extraction and fractionation of biologically active compounds from *Atraphaxis virgata*. Supercritical CO_2_ extraction was performed first to isolate lipophilic constituents, followed by ethanol–water extraction of the plant meal (solid residue after CO_2_ extraction) to recover polar metabolites (amino acids, phenolic acids, flavonoids, proteins, and carbohydrates). Subsequent solvent partitioning and chromatographic fractionation were carried out to characterize distinct metabolite classes. Fatty acids and amino acids were analyzed in both CO_2_-derived and ethanol-derived fractions to capture complementary distributions.

The extraction and initial separation steps are illustrated in the Materials and Methods (Figure 2). Figure S1 presents the GC–MS chromatogram of the CO_2_ extract from the aerial parts (stems, flowers, and leaves) of *Atraphaxis virgata*, showing multiple distinct peaks corresponding to the lipophilic mixture listed in Table 1. The most abundant peak was ethyl linoleate (peak at 36.26 min, *m*/*z* 310.5), followed by nonacosane (46.16 min, *m*/*z* 408.7) at 7.99%, palmitic acid (32.43 min, *m*/*z* 256.4) at 7.67%, and 2-nonadecanone (38.51 min, *m*/*z* 278.4) at 7.40%. Other significant constituents included heptacosane (43.25 min, *m*/*z* 380.5, 7.17%), squalene (46.35 min, *m*/*z* 408.7, 4.73%), octacosanol (41.47 min, *m*/*z* 410.7, 4.44%), and ethyl hexadecanoate (32.55 min, *m*/*z* 256.4, 4.35%). These peaks highlight the presence of diverse lipophilic compounds, including fatty acids and their esters (e.g., palmitic acid, ethyl oleate), long-chain hydrocarbons (e.g., nonacosane, heptacosane), alcohols and phytosterols (e.g., octacosanol, phytol), and bioactive terpenoids (e.g., squalene). This diversity supports the pharmacological potential of *A. virgata*, especially considering the known antioxidant, anti-inflammatory, and lipid-regulating activities of many of these compounds.

Thus, GC–MS analysis of the CO_2_ extract revealed a chemically diverse lipophilic mixture (42 identified compounds), providing the basis for comparison with the polar metabolites recovered from the ethanol–water extract of the plant meal.

### 2.2. Polar Metabolites in the Ethanol–Water Extract: Amino Acids, Proteins, and Carbohydrates (Post-CO_2_ Plant Meal) and Aqueous Residue

After GC-MS characterization of the lipophilic constituents in the CO_2_ extract, the remaining plant meal (post-CO_2_ extraction) was subjected to ultrasound-assisted ethanol–water extraction (70% EtOH, 30–35 °C, 1:8 ratio, 120 min) to recover polar metabolites. This extract was analyzed for amino acids, proteins, and carbohydrates, providing complementary information to the lipophilic profile. The free amino acid composition of the ethanol–water extract (Table 2) revealed considerable structural diversity, with proline and arginine being the most abundant, suggesting a physiological role in osmotic regulation and nitrogen metabolism. Essential amino acids such as phenylalanine and tyrosine, along with functionally important residues including methionine (sulfur-containing), histidine (heterocyclic), and hydroxylated amino acids like serine and threonine, were also detected. This pattern indicates both nutritional and bioactive potential and is consistent with profiles reported in other medicinal plants [15,16].

The protein content of *A. virgata* plant meal was determined using the Kjeldahl method, yielding 7.15%. While this value indicates moderate protein density, it is acknowledged that non-protein nitrogen sources may slightly inflate the estimate [17].

Carbohydrate profiling of the ethanol–water extract, conducted here for the first time in *A. virgata*, identified fructose as the dominant monosaccharide (47.83% of total). The disaccharide lactulose (4-O-β-D-galactopyranosyl-D-fructose) was detected at a trace level (0.6%), whereas glucose, sucrose, maltose, and lactose were below detection limits. These results align with prior studies that validate the robustness of HPLC-RID for quantifying simple sugars in complex plant-derived matrices [18].

To complement this analysis, the aqueous residue remaining after solvent-solvent partitioning was subjected to amino acid profiling by gas-liquid chromatography (GLC) on a CARLO ERBA 4200 instrument (Carlo Erba Reagents, Cornaredo, Italy). The quantitative distribution of 20 amino acids in the aqueous residue after hydrolysis is shown in Table 3. The results demonstrate that *A. virgata* contains a complex amino acid profile, with glutamic acid (2.510%) and aspartic acid (1.348%) being the most abundant, reflecting their central roles in nitrogen metabolism. In contrast, hydroxyproline (0.001%) and ornithine (0.002%) were present in minimal amounts. Together with the free amino acid profile, this complementary analysis provides both the readily available amino acid pool and the bound amino acid fraction, giving a more complete view of the nitrogenous constituents of *A. virgata*. Total protein content was also determined using the Kjeldahl method, yielding 7.15% in samples collected during the fruiting stage from the Kokpek region. This approach provided a broader picture of total amino acid distribution, including residues released upon hydrolysis.

### 2.3. Extraction Yield Assessment of A. virgata Polar Extracts

Preliminary extraction trials were conducted to assess the effect of selected parameters on yield. Among the variables tested, solvent concentration was the most influential, with 70% aqueous ethanol providing the highest recovery and therefore chosen as the extraction medium. A solid-to-solvent ratio of 1:8 at 30–35 °C was found to be practical and efficient. Extraction time trials showed that yield increased up to 60 min and then plateaued, with 120 min under ultrasound-assisted conditions proving sufficient for recovery. A second extraction step with fresh solvent did not significantly increase yield, indicating that a single cycle was adequate. The combined ethanol extracts were filtered, concentrated under reduced pressure, frozen at −20 °C, and lyophilized. The dried extract was subsequently partitioned with chloroform and ethyl acetate, with the aqueous phase retained for analysis. All extractions were carried out under these conditions, as described in Section 3.4, to maintain consistency across the workflow (Figure 2).

### 2.4. Fatty Acid Profiling of Chloroform Extracts from Atraphaxis virgata

Fatty acids were first identified in the CO_2_ extract by GC–MS, where several free and esterified forms (palmitic, oleic, linoleic, linolenic acids) were detected. To complement this, the chloroform-soluble fraction of the ethanol–water extract was also examined to assess whether residual fatty acids remained after CO_2_ extraction. The analysis confirmed the presence of a similar fatty acid spectrum, indicating that unsaturated fatty acids represent stable constituents of *A. virgata* across different extraction conditions. Fatty acid composition of the chloroform-soluble fraction was analyzed by gas–liquid chromatography using the Carlo Erba 420 system, with identification based on retention times of methyl ester standards of saturated and unsaturated fatty acids. The quantification was performed by normalization of peak areas (Table 4). The sample was rich in oleic acid (C18:1, 50.5%), linoleic acid (C18:2, 30.5%), and palmitic acid (C16:0, 11.2%), while minor constituents included myristic (C14:0, 1.0%), pentadecanoic (C15:0, 0.7%), stearic (C18:0, 5.4%), palmitoleic (C16:1, 0.1%), and linolenic (C18:3, 0.5%) acids. The lipid profile of *A. virgata* was dominated by unsaturated fatty acids, particularly oleic and linoleic acids. This pattern is consistent with recent findings in related plant species where oleic, linoleic, and palmitic acids are the principal fatty acids identified via GC–MS (e.g., in desert plants such as *G. tournefortii*) [20]. Similar trends have also been reported in herbal extracts where unsaturated acids markedly exceed saturated ones in plant lipids [21]. This fatty acid profile represents the chloroform-soluble fraction derived from the ethanol–water extraction, complementing the lipophilic composition obtained from CO_2_ extraction and thereby extending the coverage of metabolite classes.

The total lipid content of the plant meal was determined using the Soxhlet extraction method and found to be 1.11% by mass, indicating a low but biochemically significant fat fraction. Lipids and lipoids are fundamental to plant physiology, serving as structural elements of cellular membranes and storage compounds in many species. The observed prevalence of unsaturated fatty acids enhances the nutritional and bioactive potential of *A. virgata*, given their known roles in antioxidant activity and membrane stability [22].

### 2.5. Investigation of the Ethyl Acetate Extract Constituents (Structural Elucidation of Flavonoids and Phenolic Acids)

The phenolic and flavonoid constituents were isolated from the ethanol–water extract, highlighting the polar metabolite spectrum in contrast to the lipophilic compounds identified in the CO_2_ extract. Flavonoids, with their diverse structures, play important roles in plant adaptation to adverse environmental conditions, such as salt stress, as documented in earlier studies [19,23,24]. However, the occurrence of flavonoid compounds in salt-tolerant plant species remains insufficiently studied, and available data are often fragmented. In the present work, fractionation of the ethanol–water extract and subsequent purification of the ethyl acetate fraction by column chromatography on Silicagel L 100/160 yielded six phenolic compounds. Spectroscopic analyses (UV, IR, NMR, MS) confirmed four flavonol glycosides, quercetin-3-O-rutinoside (rutin), quercetin-3-O-glucoside (isoquercitrin), kaempferol-3-O-rutinoside, and isorhamnetin-3-O-glucoside, together with two phenolic acids, gallic acid and chlorogenic acid.

The flavonoid profile obtained here differs from our previously reported composition [25]. This variation is attributable to differences in plant material and developmental stage: the present study analyzed aerial parts harvested during fruiting, whereas the earlier study focused on bulk ethanolic extracts. Seasonal and phenological factors are known to influence the distribution of flavonoids in Polygonaceae species. Accordingly, the current findings extend our previous results by providing a broader phytochemical profile obtained under different ecological and developmental conditions.

Compound **1.1** was isolated as light-yellow, glossy crystals and identified as 3-O-ℒ-L-rhamnopyranoside 7-O-methyl-8-O-acetylgossypetin. Elemental analysis yielded values of C 55.04% and H 4.81%, closely aligning with the calculated values for C_24_H_24_O_13_ (C 55.38%; H 4.61%). Its flavonoid nature was initially inferred from its chromatographic mobility (paper chromatography, systems I and II), strong fluorescence under UV light, and UV absorption maxima in the range of 354–356 nm, characteristic of flavones [26]. The ^1^H NMR spectrum (CD_3_OD) exhibited a singlet at δ ~6.70 ppm corresponding to H-3, indicative of the flavone core. Additional aromatic signals included H-6 (δ ~6.20, d, J ≈ 2.0 Hz) and H-8 (δ ~6.40, d), while B-ring protons H-2′, H-5′, and H-6′ resonated at δ 7.45 (d), 7.20 (dd), and 6.90 ppm (d), respectively. Substituent-related signals were observed at δ ~3.80 ppm (OCH_3_, singlet), δ ~2.30 ppm (acetyl CH_3_), and sugar protons appeared in the δ 3.0–5.5 ppm region with a terminal rhamnose methyl at δ ~1.10 ppm (d, J ≈ 6.0 Hz). The ^13^C NMR spectrum displayed characteristic resonances: ~180 ppm (C-4), ~165 ppm (C-2), ~135 ppm (C-3, glycosylated), and signals between 150–155 ppm for C-7, C-8, and C-4′. The methoxy carbon was detected at ~56 ppm, while the acetyl methyl and carbonyl appeared at ~20 and ~170 ppm, respectively. The sugar carbons ranged from ~60 to 105 ppm, with the anomeric carbon near ~100–105 ppm and the rhamnose methyl at ~18 ppm. Infrared spectroscopy confirmed the presence of C=O and O–H functional groups with absorption bands at 1655 cm^−1^ and 3498 cm^−1^. Mass spectrometric data supported the proposed structure with a calculated molecular weight of approximately 556.46 g/mol (C_24_H_28_O_15_). The protonated molecular ion [M + H]^+^ appeared at *m*/*z* 557.46, while adducts with sodium and potassium yielded peaks at *m*/*z* 580.45 and 596.42, respectively [27]. Fragmentation patterns revealed key diagnostic losses: rhamnopyranose (164 Da), acetyl group (42 Da), and methyl group (15 Da), resulting in fragment ions at *m*/*z* 393.46, 515.46, and 542.46, respectively. A combined loss of the sugar and acetyl moiety (206 Da) produced a prominent ion at *m*/*z* 351.46. Additionally, a core fragment related to the gossypetin skeleton was observed near *m*/*z* 360, consistent with the aglycone bearing partial substituent losses. Bathochromic shifts in UV spectra upon complexation with boric acid and sodium acetate further confirmed the presence of vicinal 3′,4′-dihydroxyl groups and a free hydroxyl group at position C-7. Thus, the spectroscopic, chromatographic, and physicochemical analyses substantiate the identification of compound **1.1** as a novel flavonoid glycoside from *A. virgata*.

Compound **1.2** was isolated as an orange-yellow crystalline material and identified as 3-O-α-L-rhamnopyranoside 7-O-methylgossypetin, based on comprehensive spectral data and elemental analysis. The experimentally found values were C 54.90% and H 4.95%, which align closely with the calculated values for C_22_H_22_O_12_ (C 55.23%; H 4.60%). The compound possesses a flavone backbone bearing hydroxyl groups at positions 5, 7, 8, 3′, and 4′, with the hydroxyl at C-7 replaced by a methoxy group and a glycosidic linkage to α-L-rhamnose at position C-3. The ^1^H NMR spectrum (in CD_3_OD) showed distinct aromatic signals, including a doublet at δ ~6.20 ppm (H-6, J ≈ 2.0 Hz) and another at δ ~6.40 ppm (H-8, J ≈ 2.0 Hz), consistent with ortho-coupling within the A-ring. The B-ring signals appeared at δ 7.45 ppm (H-2′, d), δ 7.10 ppm (H-5′, dd), and δ 6.85 ppm (H-6′, d). A singlet at δ 3.80 ppm was assigned to the methoxy group at position 7. The rhamnose moiety displayed anomeric proton H-1″ at δ 5.20 ppm (d, J ≈ 1.5 Hz), with other sugar protons between δ 3.20–4.20 ppm and a methyl doublet at δ 1.10 ppm (J ≈ 6.0 Hz). In the ^13^C NMR spectrum, signals were observed at δ 180 ppm (C-4, ketone), δ 165 ppm (C-2), δ 135 ppm (C-3, glycosylated), and between δ 155–160 ppm for substituted aromatic carbons (C-5, C-7, C-8, C-9, and C-4′). The methoxy carbon appeared at δ 56 ppm, while the sugar carbons spanned from δ 60 to 105 ppm, with the anomeric carbon (C-1″) around δ 100 ppm and the methyl of rhamnose (C-6″) at δ 18 ppm. The UV spectrum revealed a maximum absorption in the range of 354–356 nm, typical for flavonol-type chromophores [26]. The IR spectrum showed characteristic peaks at 1655 cm^−1^ (C=O stretching), 3498 cm^−1^ (O–H), and in the 2900–2800 cm^−1^ region for C–H bonds associated with –OCH_3_ groups. Mass spectrometry supported these findings with a calculated molecular weight of approximately 513.42 g/mol. The protonated molecular ion appeared at *m*/*z* 514.4 [M + H]^+^, with sodium and potassium adducts observed at *m*/*z* 537.4 and 553.4, respectively. Fragmentation included a major peak at *m*/*z* 350.4 due to the loss of the rhamnopyranoside unit (164 Da), a secondary peak at *m*/*z* 499.4 from loss of a methyl group (15 Da), and a prominent gossypetin fragment carrying a methyl substituent near *m*/*z* 333 [27]. These spectroscopic and fragmentation features confirm the identity of compound **1.2** as a methylated gossypetin glycoside, a novel flavonoid derivative isolated from *A. virgata*.

Compound **1.3** was isolated as a white amorphous powder and identified as 7-O-methyl-8-O-acetylgossypetin, a non-glycosylated aglycone derivative of gossypetin (5,7,8,3′,4′-pentahydroxyflavonol). Structural features include a methoxy group at position 7 and an acetyl group at position 8, modifications frequently encountered among polymethoxylated and acylated flavonols in plant secondary metabolites [28]. In the ^1^H NMR spectrum (CD_3_OD), the aromatic protons of ring A appeared as meta-coupled doublets at approximately 6.20 ppm (H-6) and 6.40 ppm (H-8), while ring B protons were observed at ~7.45 ppm (H-2′, d), ~7.10 ppm (H-5′, dd), and ~6.85 ppm (H-6′, d), consistent with a 3′,4′-disubstituted pattern. The singlet at ~3.80 ppm was assigned to the methoxy group at C-7, and the signal at ~2.30 ppm corresponded to the methyl group of the acetyl moiety. The ^13^C NMR spectrum further confirmed the structure with resonances at ~180 ppm (C-4), ~165 ppm (C-2), and between 145 and 160 ppm for carbons bearing hydroxyl or substituted groups, including C-5, C-7, and C-8. The methoxy carbon appeared at ~56 ppm, and the acetyl methyl and carbonyl carbons were identified at ~20 ppm and ~170 ppm, respectively. High-resolution mass spectrometry showed a molecular ion [M + H]^+^ at *m*/*z* 393.3, with sodium and potassium adducts at *m*/*z* 416.3 and 432.3. Fragment ions at *m*/*z* 351.3 and 378.3 indicated losses of the acetyl and methyl groups, respectively, while a fragment at *m*/*z* 318 confirmed the presence of the gossypetin core, consistent with expected fragmentation patterns in flavonol derivatives [27]. These spectroscopic data support the identification of compound **1.3** as 7-O-methyl-8-O-acetylgossypetin, newly isolated from *A. virgata*.

Compound **1.4** was isolated as a yellow crystalline material and identified as 7-O-methylgossypetin, a methylated flavonol derivative structurally characterized as 5,7,8,3′,4′-pentahydroxyflavone bearing a methoxy substituent at position C-7. Elemental composition analysis yielded a molecular formula of C_16_H_12_O_7_, corresponding to a calculated molecular weight of approximately 316.27 g/mol. This compound lacks glycosidic and acetyl substituents, in contrast to other gossypetin derivatives identified in the extract. The ^1^H NMR spectrum in CD_3_OD showed typical aromatic signals of the flavonol core: a pair of ortho-coupled doublets at δ 6.20 ppm (H-6) and δ 6.40 ppm (H-8) with J ≈ 2.0 Hz, confirming the meta-substitution pattern in ring A, in agreement with previously described gossypetin analogues [29]. The B-ring protons were observed at δ 7.45 ppm (H-2′, d, J ≈ 2.0 Hz), δ 7.10 ppm (H-5′, dd, J ≈ 8.5, 2.0 Hz), and δ 6.85 ppm (H-6′, d, J ≈ 8.5 Hz). A singlet at δ 3.80 ppm corresponded to the 7-OCH_3_ group, while exchangeable hydroxyl signals (from OH groups at C-5, C-8, C-3′, and C-4′) were suppressed due to deuterium exchange. The ^13^C NMR spectrum further corroborated the structure, with key carbon signals including C-4 at δ ~180 ppm (C=O ketone), C-2 at δ ~165 ppm, C-3 at δ ~135 ppm, and C-7 at δ ~155 ppm (bearing the methoxy group). Aromatic carbons in ring B were found in the δ 115–150 ppm range, with the methoxy carbon appearing at δ ~56 ppm. Mass spectrometric analysis provided additional support for the proposed structure. The [M + H]^+^ ion was observed at *m*/*z* 317.3, in agreement with the monoisotopic mass of the protonated molecular ion. Adducts at *m*/*z* 339.3 ([M + Na]^+^) and 355.2 ([M + K]^+^) were also present. Fragmentation patterns were consistent with typical flavonoid ion behavior: loss of a methyl group yielded *m*/*z* 302.2 ([M + H − CH_3_]^+^), corresponding to the aglycone gossypetin; further loss of CO led to *m*/*z* 289.3, and combined CH_3_ and CO loss gave a fragment at *m*/*z* 274.2. Retro-Diels–Alder fragmentation produced characteristic signals around *m*/*z* 165 and 153, attributed to cleavage between the A- and B-ring systems. A minor peak at *m*/*z* 299.3 indicated the loss of water, likely from a phenolic OH group. The UV spectrum exhibited an absorption maximum at ~356 nm, consistent with the presence of a flavonol chromophore. The IR spectrum confirmed characteristic bands for C=O (near 1655 cm^−1^) and O–H stretching (broad band near 3498 cm^−1^). So, these spectral and structural data validate the identification of compound **1.4** as 7-O-methylgossypetin, a non-glycosylated methylated flavonoid constituent of *A. virgata*.

Among the phenolic acids, vanillic acid (4-hydroxy-3-methoxybenzoic acid) and cinnamic acid (3-phenylprop-2-enoic acid) were identified. To our knowledge, this is the first report of these acids in *Atraphaxis virgata*, expanding the documented phytochemical profile of the species. Both hydroxybenzoic and hydroxycinnamic acids have been previously reported across various plant species belonging to the *Polygonaceae* family [30]. The hydroxylation patterns of phenolic acids in *Atraphaxis* species correspond closely with the B-ring hydroxylation patterns of co-existing flavonoids identified in the same taxa—namely isorhamnetin, quercetin, and tamarixetin.

Compound **2.1** (4-hydroxy-3-methoxybenzoic acid), identified as vanillic acid, was obtained as needle-like crystals with a melting point of 213–214 °C from methanol. The compound exhibited Rf values of 0.65 and 0.45 in systems III and IV, respectively, consistent with the known chromatographic behavior of vanillic acid derivatives. Its UV absorption maximum at 272 nm (MeOH) corresponds to π → π* transitions typical for substituted benzoic acids. Elemental analysis results (C 54.22%, H 3.92%) closely matched the calculated values for C8H8O4 (C 54.55%, H 3.89%). The ^1^H NMR spectrum in CDCl_3_ (Figure S2) showed a singlet at δ 3.92–3.96 ppm for the methoxy protons (H-8), and aromatic protons H-3 and H-4/H-6 appeared as multiplets at δ 6.90–7.01 and 7.27–7.39 ppm, respectively. The hydroxyl proton H-9 was observed as a multiplet at δ 6.45–6.50 ppm, while the carboxylic acid proton H-12 appeared at δ 9.67–9.82 ppm. The corresponding ^13^C NMR spectrum (Figure S3) displayed characteristic signals at δ 56.18 ppm (C-8, methoxy), and aromatic carbons ranged from δ 108.88 to 151.89 ppm, with the carboxylic carbon (C-10) at δ ~191.17 ppm.

In the 2D ^1^H–^1^H COSY spectrum (Figure S4), scalar couplings were observed between H-3 and H-4 (δ 6.99, 7.35 ppm), consistent with vicinal proton coupling within the aromatic ring. The ^1^H–^13^C HMQC spectrum (Figure S5) confirmed direct proton-carbon one-bond correlations, including H8–C8 (3.90, 56.36 ppm) and H3–C3 (7.00, 114.69 ppm). The ^1^H–^13^C HMBC spectrum (Figure S6) demonstrated long-range correlations such as H8–C1 (3.90, 147.73 ppm), H9–C3 (6.49, 114.53 ppm), and H12–C4/C6 (9.78, 130.24 and 109.37 ppm), supporting the structure of the substituted benzoic acid ring system.

The IR spectrum (Figure S7) confirmed the presence of broad O–H stretching near 3400 cm^−1^ and strong C=O stretching at approximately 1700 cm^−1^, typical of carboxylic acids. Collectively, these data conclusively validate compound **2.1** as vanillic acid, a known phenolic acid previously reported in many *Polygonaceae* species and now confirmed in *A. virgata*. The structural relation between this compound and the B-ring hydroxylation pattern observed in flavonoids such as quercetin and isorhamnetin suggests coordinated biosynthetic pathways within the plant.

Compound **2.2** (Cinnamic Acid (3-Phenylprop-2-enoic acid)) was isolated as a crystalline solid and identified as cinnamic acid based on a comprehensive set of spectral analyses, including ^1^H and ^13^C NMR, 2D NMR (COSY and HMQC), and IR spectroscopy. The ^1^H NMR spectrum (Figure S8) revealed characteristic signals for an aromatic system and a trans-alkene moiety. The doublets observed at δ 7.35 ppm and δ 6.35 ppm with coupling constants J ≈ 15.9 Hz confirm the presence of a trans configuration across the double bond (H-7 and H-8), which is diagnostic for cinnamic acid derivatives. The aromatic proton multiplets between δ 7.15–7.55 ppm further corroborate the phenyl substitution pattern. Correspondingly, the ^13^C NMR spectrum (Figure S9) displayed signals at δ ~146.5 ppm (C-7), δ ~117.3 ppm (C-8), and δ ~167.5 ppm (C-9, carboxylic group), in agreement with literature values for cinnamic acid structures. The reliability of such assignments underscores the central role of NMR-based characterization in phytochemical studies, consistent with recent applications of NMR for structural validation of plant-derived inclusion complexes [31].

Further confirmation was obtained from 2D NMR experiments. The ^1^H–^1^H COSY spectrum (Figure S10) showed clear scalar coupling between H-7 and H-8, reinforcing the trans-stereochemistry of the double bond. In the ^1^H–^13^C HMQC spectrum (Figure S11), direct correlations were observed between proton and carbon resonances, validating the protonated carbon assignments and confirming the proposed carbon framework.

The IR spectrum of compound **2.2** (Figure S12) supported the presence of conjugated carboxylic acid functionality, as indicated by a strong absorption band at ~1680 cm^−1^ (C=O stretch) and a broad band around ~3100–3300 cm^−1^ attributable to the –OH group of the carboxylic acid. Additional peaks at ~1600 and ~1500 cm^−1^ confirmed the aromatic skeletal vibrations.

The assignment of compound **2.2** as cinnamic acid is consistent with known phytochemical profiles of *Atraphaxis virgata*, particularly in relation to the presence of hydroxycinnamic acid derivatives commonly reported in the *Polygonaceae* family. The spectral characteristics and the molecular formula support the identification of 3-phenylprop-2-enoic acid without additional hydroxyl or methoxy substitutions.

### 2.6. Determination of Antioxidant Content

To date, over 6000 naturally occurring plant-derived antioxidants are recognized, primarily classified as flavonoids. These compounds share a common C6–C3–C6 backbone and exhibit strong radical-scavenging activity due to their hydroxyl substituents. The antioxidant potential of flavonoids is closely correlated with the number and arrangement of hydroxyl groups on the aromatic rings, influencing their redox properties and biological effects [32,33]. The antioxidant profile of each plant species varies with factors such as phenological stage, geographic origin, and local environmental conditions such as temperature, soil composition, and sunlight exposure [11,34,35,36]. Therefore, before designating a plant as a source of bioactive antioxidants, it is essential to evaluate its antioxidant capacity, clearly noting the geographical origin and timing of harvest. Recent findings by Shin et al. [9] demonstrated that *A. virgata* exhibits considerable in vitro antioxidant activity, including ABTS, DPPH, and FRAP radical-scavenging capacities, which were attributed to the presence of flavonoids such as kaempferol, rutin, and hesperidin in the aerial parts of the plant. These results support the relevance of further examining the antioxidant composition and potency of *A. virgata* from different regions. Table 5 presents the results of total antioxidant content (TAC) measurements in *Atraphaxis virgata* extracts. Quercetin was used as the standard reference compound in all analyses. Antioxidant activity was evaluated separately in the lipophilic CO_2_ extract and in the polar ethanol–water extract of the plant meal, corresponding to lipid- and water-soluble antioxidant fractions, respectively (Table 5).

The data indicate that the water-soluble antioxidants are present in significantly higher concentrations in the aerial parts of *A. virgata* compared to the lipid-soluble fraction. All antioxidant assays were performed in triplicate. Results are expressed as te mean ± SD. Statistical significance between lipid- and water-soluble antioxidant fractions was assessed using one-way ANOVA with Tukey’s post hoc test (*p* < 0.05 considered significant).

### 2.7. Immunomodulatory Activity of A. virgata

It is well established that immune system dysregulation accompanies the majority of pathological conditions, particularly in response to cytotoxic stress or inflammation [37]. In this study, the immunomodulatory properties of the hydroethanolic extract of *Atraphaxis virgata* were investigated in healthy, sexually mature laboratory rats to assess its stimulatory effects on erythropoiesis, leukopoiesis, and thrombopoiesis.

Administration of the cytostatic agent cyclophosphamide significantly suppressed hematopoiesis, resulting in marked reductions across all parameters of peripheral blood. Erythrocyte counts declined by 1.95-fold (*p* < 0.01), accompanied by a 1.53-fold decrease in hemoglobin levels (*p* < 0.05). Platelet counts dropped by 1.64-fold, and total leukocyte counts were reduced by 2.34-fold (*p* < 0.01), indicating systemic suppression of hematopoietic activity.

Intramuscular administration of the *A. virgata* extract in rats with experimentally induced erythro-, leuko-, and thrombocytopenia, hematological analyses revealed moderate but statistically significant recovery in several parameters (Table 6). The erythrocyte count increased from 3.59 ± 0.20 × 10^12^/L to 6.92 ± 0.02 × 10^12^/L (*p* < 0.01), representing a 1.92-fold restoration. Hemoglobin levels improved from 96.0 ± 2.67 g/L to 122.0 ± 21.22 g/L (*p* < 0.05), and hematocrit values rose from 20.75 ± 0.30% to 32.09 ± 3.25% (*p* < 0.05). Mean corpuscular volume (MCV) increased significantly from 41.8 ± 0.07 fL to 46.0 ± 4.54 fL (*p* < 0.05), indicating erythroid regeneration.

Following ethanol–water extraction and ultrasound-assisted preparation, the hydroethanolic fraction was administered intramuscularly for five consecutive days to cyclophosphamide-treated Wistar rats. This regimen produced a marked recovery of leukocyte indices, with total leukocyte counts rising from 4.72 ± 0.35 × 10^9^/L to 12.32 ± 2.22 × 10^9^/L (*p* < 0.01). Lymphocyte percentages increased from 57.01 ± 1.65% to 92.5 ± 4.24% (*p* < 0.05), and neutrophil counts rose from 1.70 ± 0.60 × 10^9^/L to 11.39 ± 0.84 × 10^9^/L (*p* < 0.01). These findings demonstrate that the administered extract promoted leukopoietic and erythropoietic recovery under cytostatic stress. In contrast, platelet counts showed no significant recovery (*p* > 0.05), indicating that thrombopoiesis was minimally influenced under the given experimental conditions. Together, these findings suggest that the *A. virgata* extract exerts moderate immunorestorative effects, particularly in the stimulation of leukopoiesis and, to a lesser extent, erythropoiesis, while showing minimal influence on thrombopoietic recovery. This steady hematopoietic stimulation profile highlights the extract’s potential as a supportive agent in conditions of myelosuppression.

Recent studies on natural compounds with immunomodulatory potential support such findings. For instance, plant-derived flavonoids and polyphenols have been shown to stimulate hematopoietic recovery and modulate cytokine balance in immunocompromised models [10,38]. Moreover, certain botanical extracts have demonstrated similar effects on leukocyte proliferation and erythropoietic regeneration following chemotherapeutic damage [39].

Flavonoids and phenolic acids, which were identified in *A. virgata*, have been reported in other Polygonaceae species to exert antioxidant and immunomodulatory effects. This broader evidence supports the biological plausibility of the activities observed in *A. virgata* extracts and demonstrates consistency with known pharmacological properties of Polygonaceae metabolites.

## 3. Materials and Methods

### 3.1. Plant Material

Aerial parts of *Atraphaxis virgata* were collected during the fruiting stage in July 2021 near Kokpek village (Enbekshikazakh district, Almaty region, Kazakhstan) (Figure 1). The species was authenticated by Dr. N.G. Gemedzhieva (Institute of Botany and Phytointroduction, Almaty, Kazakhstan). A voucher specimen (AV2021-07) was deposited in the herbarium of the institute.

### 3.2. General Analytical Procedures

UV spectra were recorded on a diode-array detector (YL Instruments, Anyang-si, Republic of Korea). FT-IR spectra were recorded on a Shimadzu IRTracer-100 instrument (Kyoto, Japan), NMR spectra on a Bruker Avance III 400 MHz (TMS internal standard), and melting points using a Stuart SMP30 apparatus. GC–MS analyses were performed on an Agilent 7890B GC coupled to a 5977A MSD with an HP-5MS column. All solvents and standards (Sigma-Aldrich, St. Louis, MO, USA) were of analytical grade. After CO_2_ extraction, the resulting plant meal was extracted with 70% ethanol (120 min, 30–35 °C) to obtain the ethanol–water extract (56 g). This extract was subjected to compositional analyses (amino acids, protein, carbohydrates) and then partitioned sequentially into chloroform, ethyl acetate, and aqueous fractions for further phytochemical investigation, as summarized in Figure 2.

A schematic overview of the full experimental workflow, including extraction, fractionation, and analytical procedures applied in this study, is presented in Figure 2.

### 3.3. Supercritical CO_2_ Extraction

Dried aerial parts were extracted using an SFE-500 system (Thar Instruments, Pittsburgh, PA, USA) at 55 °C, 200 bar, and 120–180 min. The procedure yielded a lipophilic fraction enriched in fatty acids, sterols, hydrocarbons, and terpenoids, which was subjected to GC–MS analysis. This extraction was performed in parallel with an ethanol–water protocol (Section 3.4) to provide complementary access to polar constituents. The two approaches were not intended for direct yield comparison but for comprehensive metabolite coverage across polarity classes.

### 3.4. Ultrasound-Assisted Ethanol–Water Extraction (UAE) of the Plant Meal

To complement the CO_2_ extraction of lipophilic metabolites, the residual plant meal was subjected to water–ethanol (70%) extraction with ultrasound stimulation at 30–35 °C, using a solid-to-solvent ratio of 1:8 for 120 min. The combined extracts were filtered, concentrated under reduced pressure, and freeze-dried. The dried extract was then partitioned with chloroform, ethyl acetate, and water to yield fractions for compositional and phytochemical analyses (Figure 2). These fractions were further evaluated in vivo. In a cyclophosphamide-induced myelosuppression model in Wistar rats, the hydroethanolic extract was administered intramuscularly for five consecutive days. Hematological analysis of blood samples collected three days after treatment included leukocyte, erythrocyte, and platelet indices. This workflow established a direct link between the extraction methodology and the observed immunomodulatory effects.

### 3.5. Fatty Acid Analysis

Fatty acid composition was determined both in the CO_2_ extract and in the chloroform fraction obtained after solvent–solvent partitioning of the ethanol–water extract. This dual approach allowed evaluation of free lipophilic fatty acids captured directly by CO_2_ extraction and residual fatty acids released into the chloroform fraction following ethanol–water extraction of the plant meal. The chloroform-soluble fraction was derivatized to fatty acid methyl esters (FAMEs) and analyzed by GC–FID/GC–MS. Identification was based on authentic FAME standards and the NIST spectral library. Lipid content was quantified by means of Soxhlet extraction.

### 3.6. Amino Acid and Protein Determination

Free amino acids were analyzed using capillary electrophoresis (Kapel-105, Lumex, Saint Petersburg, Russia) with a 20 mM borate buffer (pH 9.2) at 254 nm, and identification confirmed by comparison with standards. The amino acid analysis followed our previously reported procedure [12], with minor modifications to adapt it for the present study. This ensured methodological consistency while allowing expanded profiling and integration with the current phytochemical and biological assessments.

Total protein was quantified using the Kjeldahl method (AOAC 978.04; N × 6.25). Carbohydrate content was determined by means of HPLC with refractive index detection.

### 3.7. Phenolic and Flavonoid Analysis

Phenolic and flavonoid constituents were analyzed in the ethanol–water extract and its solvent-partitioned fractions (ethyl acetate, aqueous). Chromatographic separation was carried out using an Agilent 1260 Infinity HPLC (Santa Clara, CA, USA) system equipped with a Zorbax Eclipse Plus C18 column (250 × 4.6 mm, 5 μm). The mobile phase consisted of solvent A (0.1% formic acid in water) and solvent B (acetonitrile) with a gradient elution from 10% to 90% B over 60 min at a flow rate of 1.0 mL/min. Detection was performed at 280 nm and 340 nm. Identification was based on comparison with authentic reference standards (quercetin, kaempferol, gallic acid, chlorogenic acid).

### 3.8. Isolation and Structural Elucidation of Polyphenolic Compounds

The ethyl acetate fraction of the ethanol–water extract (residue after CO_2_ extraction) was subjected to open-column chromatography on Silica gel L 100/160 (Merck, Darmstadt, Germany). Gradient elution was performed using solvent systems of increasing polarity (chloroform → chloroform–methanol mixtures) to separate flavonoid glycosides and phenolic acids. Fractions were collected and monitored by thin-layer chromatography (TLC) under UV light (254/365 nm) with AlCl_3_ spraying for flavonoid detection. Fractions with similar chromatographic profiles were pooled, concentrated under reduced pressure, and further purified by recrystallization or repeated column chromatography where necessary. The isolated compounds were characterized using a combination of UV-Vis spectroscopy, FT-IR, ^1^H and ^13^C NMR, 2D NMR (COSY, HSQC, and HMBC), and mass spectrometry. Identification was based on comparison with published data and authentic standards where available (quercetin, kaempferol, gallic acid, chlorogenic acid). This procedure yielded several flavonol glycosides and phenolic acids.

### 3.9. Antioxidant Activity

Total antioxidant content was determined using quercetin as a reference standard. Lipid- and water-soluble antioxidant fractions were quantified spectrophotometrically. Statistical comparisons were made using one-way ANOVA with Tukey’s post hoc test.

### 3.10. Immunomodulatory Activity Assessment

The in vivo immunomodulatory activity of the *A. virgata* hydroethanolic extract was evaluated in a cyclophosphamide-induced myelosuppression model using sexually mature Wistar rats of both sexes (10–15 weeks, 210–280 g; *n* = 8 per group). Animals were obtained from the animal facility of the Biological Clinic, Faculty of Biology and Biotechnology, Al-Farabi Kazakh National University, and acclimatized for one week under standard laboratory conditions (22 ± 2 °C, 12 h light/dark cycle, standard pellet diet, and water ad libitum). Body weight variation within groups did not exceed ±10%.

To minimize circadian variability, all procedures and blood collection were performed at 09:00 h. Rats were randomly divided into four groups: (1) intact control, (2) cyclophosphamide control, (3) cyclophosphamide + *A. virgata* extract, and (4) cyclophosphamide + placebo. Myelosuppression was induced by a single intraperitoneal injection of cyclophosphamide sodium (250 mg/kg). Starting 24 h later, the extract was administered intramuscularly at 100 mg/kg (0.5 mL per rat, dissolved in physiological saline) once daily for five consecutive days. The placebo group received saline on the same schedule.

Three days after the final administration, rats were briefly anesthetized with diethyl ether vapor, and blood was collected from the orbital sinus into EDTA-coated tubes. Hematological indices were measured using an automated veterinary hematology analyzer (Abacus Junior Vet, Diatron, Budapest, Hungary). The following parameters were evaluated to assess hematopoietic recovery:

- Leukocyte profile: WBC, lymphocytes (LYM, %, ×10^9^/L), neutrophils (NEU, %, ×10^9^/L), and monocytes (MON, %, ×10^9^/L)
- Erythrocyte profile: RBC, HGB, HCT, MCV, MCH, MCHC, and RDW
- Platelet profile: PLT, PCT, MPV, and PDW

These markers were selected to monitor the recovery of leukopoiesis, erythropoiesis, and thrombopoiesis following cytostatic challenge.

### 3.11. Ethical Compliance and Chronobiological Design for Immunomodulatory Activity Assessment

The study of immunomodulatory activity was conducted in accordance with chronobiological principles and in full compliance with ethical standards for the use of laboratory animals, as outlined in the Order of the Minister of Health of the Republic of Kazakhstan dated 4 November 2020, No. KR DSM-181/2020, “On approval of the rules for the assessment of materials and compliance of preclinical (non-clinical) studies with Good Laboratory Practice (GLP) requirements of the Republic of Kazakhstan and/or the Eurasian Economic Union within the framework of pharmaceutical inspection” (registered with the Ministry of Justice of the Republic of Kazakhstan on 5 November 2020 under No. 21596). https://adilet.zan.kz/eng/docs/V2000021596 (accessed on 12 September 2024).

### 3.12. Statistical Analysis

Statistical analyses were applied to both antioxidant assays and hematological data from the immunomodulatory study. Antioxidant assays were performed in triplicate, and results are expressed as the mean ± standard deviation (SD). Differences in antioxidant content were assessed using one-way analysis of variance (ANOVA) with Tukey’s post hoc test in SPSS Statistics version 26.0 (IBM Corp., Armonk, NY, USA).

For immunological data, analyses were performed in GraphPad Prism 9.0 (GraphPad Software, San Diego, CA, USA). Normality was tested using the Shapiro–Wilk test, and group differences were evaluated by one-way ANOVA followed by Tukey’s multiple comparison test. Statistical significance was set at *p* < 0.05. Levels of significance are indicated in the text, tables, and figures as *p* < 0.05, *p* < 0.01, and *p* < 0.001 versus the cyclophosphamide control group.

All sample collection and analyses were conducted at 09:00 h to minimize circadian variability in hematological indices.

Our findings suggest that several flavonoids and phenolic acids in *Atraphaxis virgata* may influence pathways linked to anxiety. Quercetin and kaempferol derivatives have been shown to enhance GABAergic signaling, producing calming effects. Cinnamic and vanillic acids act as antioxidants and reduce inflammation, both of which are strongly tied to anxiety states. Some flavonoid glycosides also interact with serotonergic transmission, offering another plausible route of action. While these mechanisms provide a biological basis for the observed effects, certain limitations must be recognized. The study used a single myelosuppression model that does not capture behavioral signs of anxiety. The experiments also lacked a positive control drug, such as diazepam, which would allow comparison with an established anxiolytic standard. Future studies should employ validated anxiety models and include benchmark drugs to better define the therapeutic relevance of *A. virgata*.

## 4. Conclusions

The findings of this study highlight *Atraphaxis virgata* as a promising botanical resource with a broad repertoire of bioactive constituents. The CO_2_ extract contained diverse lipophilic compounds, including unsaturated fatty acids, sterols, and terpenoids, while ethanol–water extracts yielded flavonol glycosides and phenolic acids with strong antioxidant potential. The amino acid profile of the defatted plant meal further underscored nutritional relevance, with the presence of both essential and conditionally essential residues. Antioxidant assays confirmed a higher abundance of water-soluble antioxidants, consistent with the prevalence of phenolic metabolites. Importantly, biological evaluation in a cyclophosphamide-induced myelosuppression model demonstrated stimulation of erythropoiesis and leukopoiesis, supporting immunomodulatory potential.

Altogether, this work provides the first comprehensive phytochemical and biological assessment of *A. virgata*, extending beyond prior fragmentary reports on the genus. By establishing chemical diversity alongside evidence of biological activity, this study lays a foundation for mechanistic exploration, pharmacological validation, and the potential development of *A. virgata* as a plant-based medicinal resource.

## Figures and Tables

**Table 1:** Lipophilic constituents of the CO_2_ extract of *A. virgata* (GC–MS analysis).

No.	Compound	Molecular Weight	Retention Time, min	Identification Probability, %	Relative Content, %
1	Heneicosane	296.58	15.00	71	0.32
2	Tetradecane	198.39	12.75	78	0.33
3	2,6,10-Trimethyl-tetradecane	240.5	21.39	63	0.26
4	5,9-undecadien-2-one, 6,10-Dimethyl-, (Z)	190.31	21.65	62	0.56
5	Hexadecane	226.44	22.49	83	0.43
6	3-Hydroxy-4-methoxybenzaldehyde	152.15	23.33	61	0.73
7	Vanillin	152.15	23.41	65	1.06
8	5-Pentyl-1,3-benzenediol	104	24.85	61	0.85
9	(R)-2(4H)-Benzofuranone, 5,6,7,7a-tetrahydro-4,4,7a-trimethyl-	213.37	26.44	72	1.45
10	Fumaric acid, 2-chlorophenylethyl ester	116.07	26.52	71	0.85
11	Octadecane	254.49	27.05	82	0.81
12	3,4,5-Trimethoxybenzaldehyde	196.20	28.23	72	0.40
13	6,10,14-Trimethyl-2-pentadecanone	268.5	29.49	71	1.53
14	Heneicosane	296.57	29.73	77	1.21
15	3-(1-Hydroxy-1-methylethyl)-5-methoxy-1,2,3,4-tetrahydro-2-naphthalenol	222.28	30.59	70	0.63
16	2-Heptadecanone	254.44	31.05	74	0.95
17	Eicosane	282.55	31.19	82	0.83
18	2-Methyl-1-hexadecanol	258.47	31.36	63	0.85
19	(E,E)-6,10,14-Trimethyl-5,9,13-pentadecatrien-2-one	290.50	32.09	73	0.41
20	Palmitic acid	256.42	32.43	78	7.67
21	Ethyl hexadecanoate	256.43	32.55	86	4.35
22	3-Ethyl-5-(2-ethylbutyl)octadecane	284.48	32.76	63	1.27
23	Heneicosane	354.68	33.13	87	1.93
24	Dibutyl phthalate	282.54	34.55	91	1.71
25	Phytol	278.34	34.83	82	2.95
26	2-Nonadecanone	296.53	34.93	88	5.14
27	Ethyl oleate	389.50	36.08	84	4.06
28	Ethyl 9,12-octadecadienoate	310.5	36.26	79	10.66
29	Ethyl 9,12,15-octadecatrienoate	308.5	36.62	71	2.18
30	2-Nonadecanone	278.44	38.51	79	7.40
31	4,8,12,16-Tetramethylheptadecan-4-olide	268.52	40.29	72	1.43
32	Octacosanol	410.73	41.47	80	4.44
33	2-Nonadecanone	410.75	41.81	68	1.86
34	Heptacosane	268.52	43.25	91	7.17
35	Tetratetracontane	380	43.53	60	0.40
36	Bis(2-ethylhexyl) phthalate	478	43.78	74	1.26
37	Octacosane	390.55	41.47	78	1.47
38	17-Pentatriacontene	394	44.90	69	1.33
39	Nonacosane	492.96	46.16	91	7.99
40	Squalene	408.79	46.36	92	4.73
41	Hentriacontane	410.73	48.94	80	2.89
42	Triacontanoic acid	436.85	52.11	60	1.26
	Total				98.25

Identification probability (%) refers to the library match score obtained from the NIST MS database. Molecular weights are derived from reference spectra in the NIST database or standard chemical references. Content (%) is expressed as the relative proportion of each compound based on peak area normalization. The results are presented as the mean values from six aliquots (*n* = 6) per sample, with <5% variation between replicates. Molecular weights are given where available.

**Table 2:** Free amino acid composition of the ethanol–water extract (from the solid residue after CO_2_ extraction) of *A. virgata*. Values are expressed as mg/100 g dry weight (mean ± SD, *n* = 3).

No.	Amino Acid	Conc., mg/100 g
1	Arginine (Arg)	87.0
2	Lysine (Lys)	20.0
3	Tyrosine (Tyr)	24.0
4	Phenylalanine (Phe)	50.0
5	Histidine (His)	18.0
6	Leucine + Isoleucine (Leu + Ile)	59.0
7	Methionine (Met)	22.0
8	Valine (Val)	43.0
9	Proline (Pro)	150.0
10	Threonine (Thr)	42.0
11	Serine (Ser)	45.0
12	Alanine (Ala)	30.0
13	Glycine (Gly)	33.0

**Table 3:** Amino acid composition of *A. virgata* extracts.

No.	Amino Acid	Abbreviation	Content, %
1	Alanine	Ala	0.292
2	Glycine	Gly	0.380
3	Leucine	Leu	0.320
4	Isoleucine	Ile	0.280
5	Valine	Val	0.274
6	Glutamic acid	Glu	2.510
7	Threonine	Thr	0.263
8	Proline	Pro	0.506
9	Methionine	Met	0.074
10	Serine	Ser	0.345
11	Aspartic acid	Asp	1.348
12	Cystine	Cys	0.020
13	Hydroxyproline	O-prp	0.001
14	Phenylalanine	Phe	0.292
15	Tyrosine	Tyr	0.334
16	Histidine	His	0.200
17	Ornithine	Orn	0.002
18	Arginine	Arg	0.542
19	Lysine	Lys	0.305
20	Tryptophan	Trp	0.080

Values represent the percentage of each amino acid relative to the total amino acid content. Amino acid analysis was performed as described in Section 3.1 using a Carlo Erba amino acid analyzer (Italy). Some components have been reported previously [19]; the current results extend this dataset with additional identified fatty acids and integration into the broader phytochemical profile.

**Table 4:** Fatty acid composition of *A. virgata*.

No.	Fatty Acid Index	Fatty Acid Name	Content (%)
1	C14:0	Myristic acid	1.0
2	C15:0	Pentadecanoic acid	0.7
3	C16:0	Palmitic acid	11.2
4	C16:1	Palmitoleic acid	0.1
5	C18:0	Stearic acid	5.4
6	C18:1	Oleic acid	50.5
7	C18:2	Linoleic acid	30.5
8	C18:3	Linolenic acid	0.5

Fatty acids were identified and quantified by means of gas–liquid chromatography on a CARLO ERBA 420 GC system (Carlo Erba Instruments, Italy) under the conditions described in Section 3.1. General Experimental Procedures.

**Table 5:** Total antioxidant content in lipid-soluble (CO_2_ extract) and water-soluble (ethanol–water extract) fractions of *A. virgata*.

No.	Parameter	Percentage (%)
1	Lipid-soluble antioxidant content	1.78 ± 0.03
2	Water-soluble antioxidant content	3.15 ± 0.04

Values are presented as the mean ± SD (*n* = 3). Statistical analysis was performed by one-way ANOVA with Tukey’s post hoc test; differences between lipid- and water-soluble antioxidant content were statistically significant (*p* < 0.05).

**Table 6:** Peripheral blood indices following cyclophosphamide-induced myelosuppression and treatment with *A. virgata* extract.

	*A*. *virgata* Extract Group	Control (Cyclophosphamide) Group	Placebo Group	Intact (Untreated) Group
WBC, ×10^9^/L	12.32 ± 2.22 **	6.20 ± 0.47	4.72 ± 0.35	11.08 ± 0.32
LYM, %	92.5 ± 4.24 *	60.04 ± 3.93	57.01 ± 1.65	69.72 ± 1.1
NEU, %	3.5 ± 0.03	32.68 ± 1.6	36.05 ± 9.3	30 ± 0.8
MI, %	3.9 ± 0.01	7.28 ± 0.4	7.03 ± 5.3	0.28 ± 0.1
LYM, ×10^9^/L	0.44 ± 0.00	3.73 ± 0.30	2.69 ± 0.87	7.72 ± 1.03
NEU, ×10^9^/L	11.39 ± 0.84 **	2.02 ± 0.91	1.70 ± 0.6	3.32 ± 0.72
MON, ×10^9^/L	0.49 ± 0.01	0.45 ± 0.00	0.33 ± 0.00	0.03 ± 0.00
RBC, ×10^12^/L	6.92 ± 0.02 **	6.06 ± 0.06	3.59 ± 0.20	7.02 ± 0.23
HGB, g/L	122 ± 21.22 *	125 ± 4.00	96 ± 2.67	147 ± 6.00
HCT, %	32.09 ± 3.25 *	23.35 ± 0.70	20.75 ± 0.30	37.3 ± 0.27
MCV, fL	46 ± 4.54 *	54.45 ± 0.43	41.8 ± 0.07	82.6 ± 0.23
MCH, pg	17.6 ± 4.24	12.75 ± 0.43	12.25 ± 0.30	18.4 ± 0.17
MCHC, g/L	379 ± 122.24	428 ± 9.33	363.6 ± 5.00	406 ± 4.00
RDWC, %	23.6 ± 2.88	25.35 ± 0.57	23 ± 0.40	23.6 ± 0.20
PLT, ×10^9^/L	382 ± 45.87	521 ± 135.33	422 ± 41.33	690 ± 166.33
PCT, %	0.27 ± 0.01	0.2815 ± 0.07	0.23 ± 0.02	0.372 ± 0.08
MPV, fL	7.1 ± 0.02	6.1 ± 0.47	5.5 ± 0.13	7.4 ± 0.30
PDWS, %	10.1 ± 0.51	12.25 ± 0.57	11.25 ± 0.23	11.4 ± 0.43

Values are presented as the mean ± standard deviation (SD), *n* = 8 rats per group. Statistical analysis was performed using one-way ANOVA followed by Tukey’s post hoc test. Significance levels: * *p* < 0.05, ** *p* < 0.01 vs. cyclophosphamide control group.

## Data Availability

Data are contained within the article.

## Supplementary Materials

The following supporting information can be downloaded at https://www.mdpi.com/article/10.3390/ijms262110301/s1.

## Abbreviations

The following abbreviations are used in this manuscript:

GC–MS	Gas Chromatography–Mass Spectrometry
HPLC	High-Performance Liquid Chromatography
UV-Vis	Ultraviolet–Visible Spectroscopy (UV-Vis)
IR	Infrared Spectroscopy
NMR	Nuclear Magnetic Resonance Spectroscopy
MS	Mass Spectrometry
HPLC-DAD	High-Performance Liquid Chromatography with Diode Array Detection
LC-MS	Liquid Chromatography–Mass Spectrometry
UAE	Ultrasound-Assisted Extraction
GLC	Gas-Liquid Chromatography
BA	Biochemical Assays
TLC	Thin-Layer Chromatography
PC	Paper Chromatography
^1^H NMR	Proton Nuclear Magnetic Resonance Spectroscopy
^13^C NMR	Carbon-13 Nuclear Magnetic Resonance Spectroscopy
^1^H–^1^H COSY	Proton–Proton Correlation Spectroscopy
^1^H–^13^C HMQC	Heteronuclear Multiple Quantum Coherence
^1^H–^13^C HMBC	Heteronuclear Multiple Bond Correlation
