# Supplementary figures and images for: Biologically Active Compounds of Plants of the Atraphaxis Genus: Chemical Composition and Immunomodulatory Evaluation
Source: Int J Mol Sci. 2025 Oct 23;26(21):10301. doi: 10.3390/ijms262110301 (PMC12610063; doi:10.3390/ijms262110301)

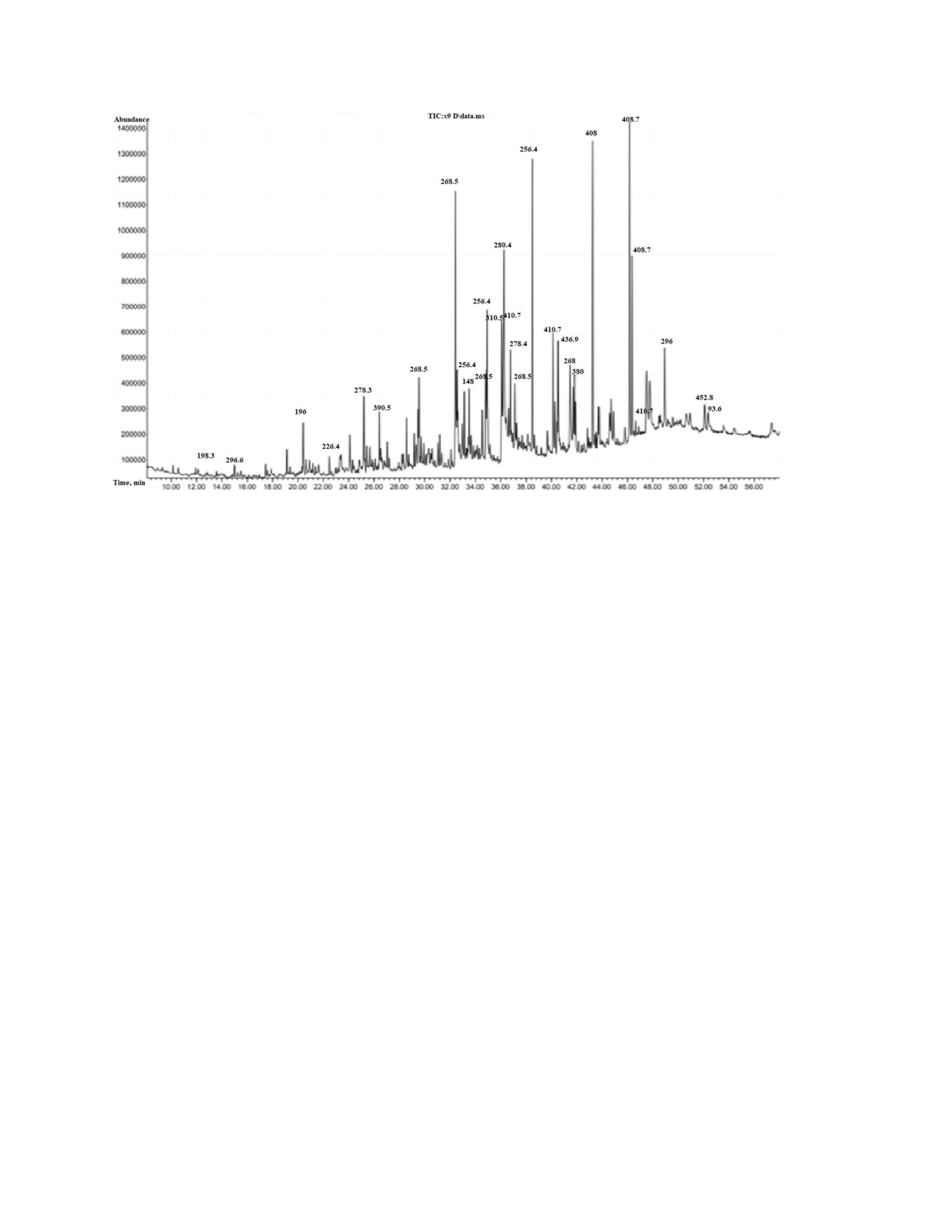

Supplement: Supplementary file 1 [file ijms-26-10301-s001.zip › Figure S1. GC−MS chromatogram of the aerial parts (stems, flowers, and leaves) of A.virgata CO2 extract..jpg]

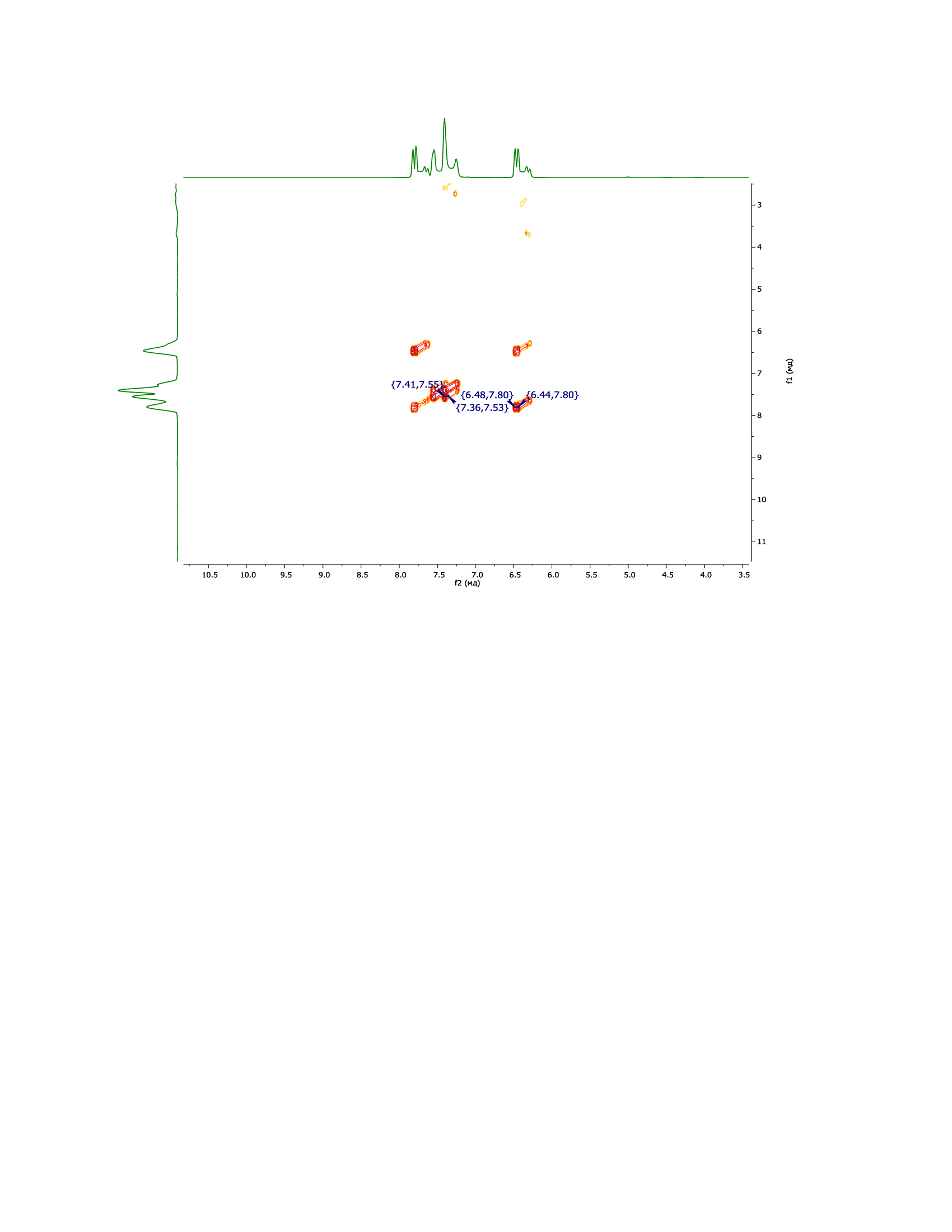

Supplement: Supplementary file 1 [file ijms-26-10301-s001.zip › Figure S10.-2D-COSY-_Correlation-Spectroscopy_-spectrum-of-compound-2.2-recorded-in-CD3OD..jpg]

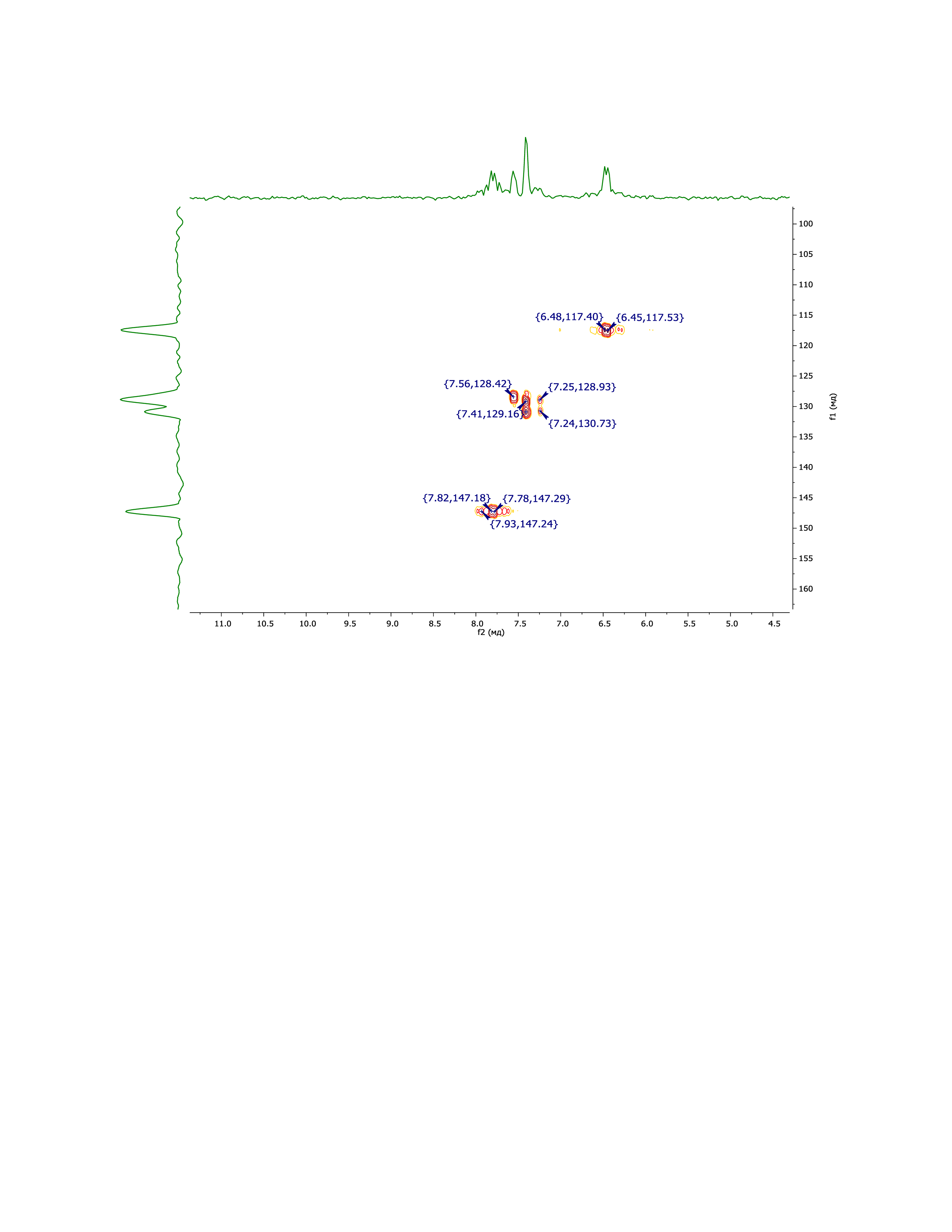

Supplement: Supplementary file 1 [file ijms-26-10301-s001.zip › Figure S11. 2D 1H–13C HMQC (Heteronuclear Single Quantum Coherence) spectrum of compound 2.2 recorded in CD3OD..jpg]

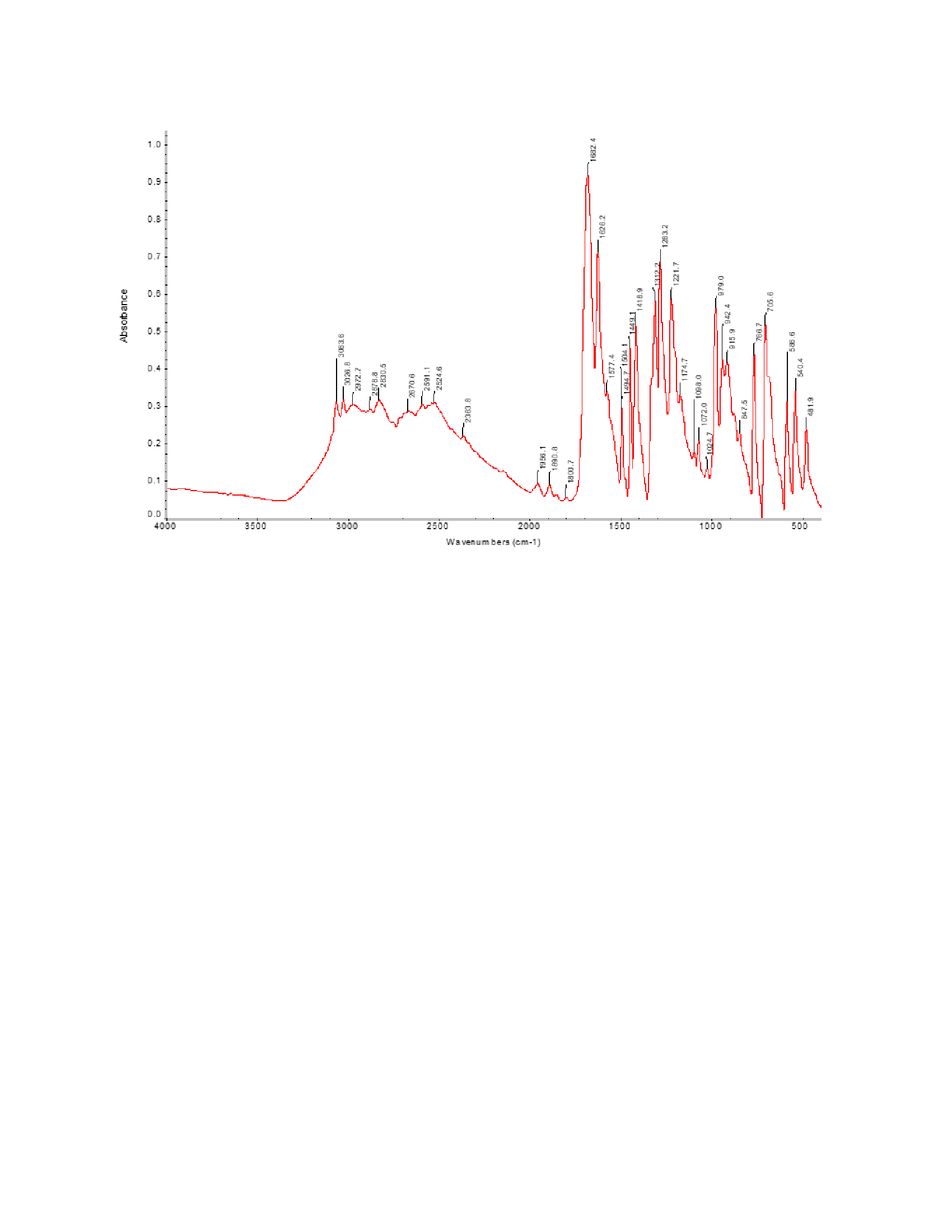

Supplement: Supplementary file 1 [file ijms-26-10301-s001.zip › Figure S12.-FT-IR-spectrum-of-compound-2.jpg]

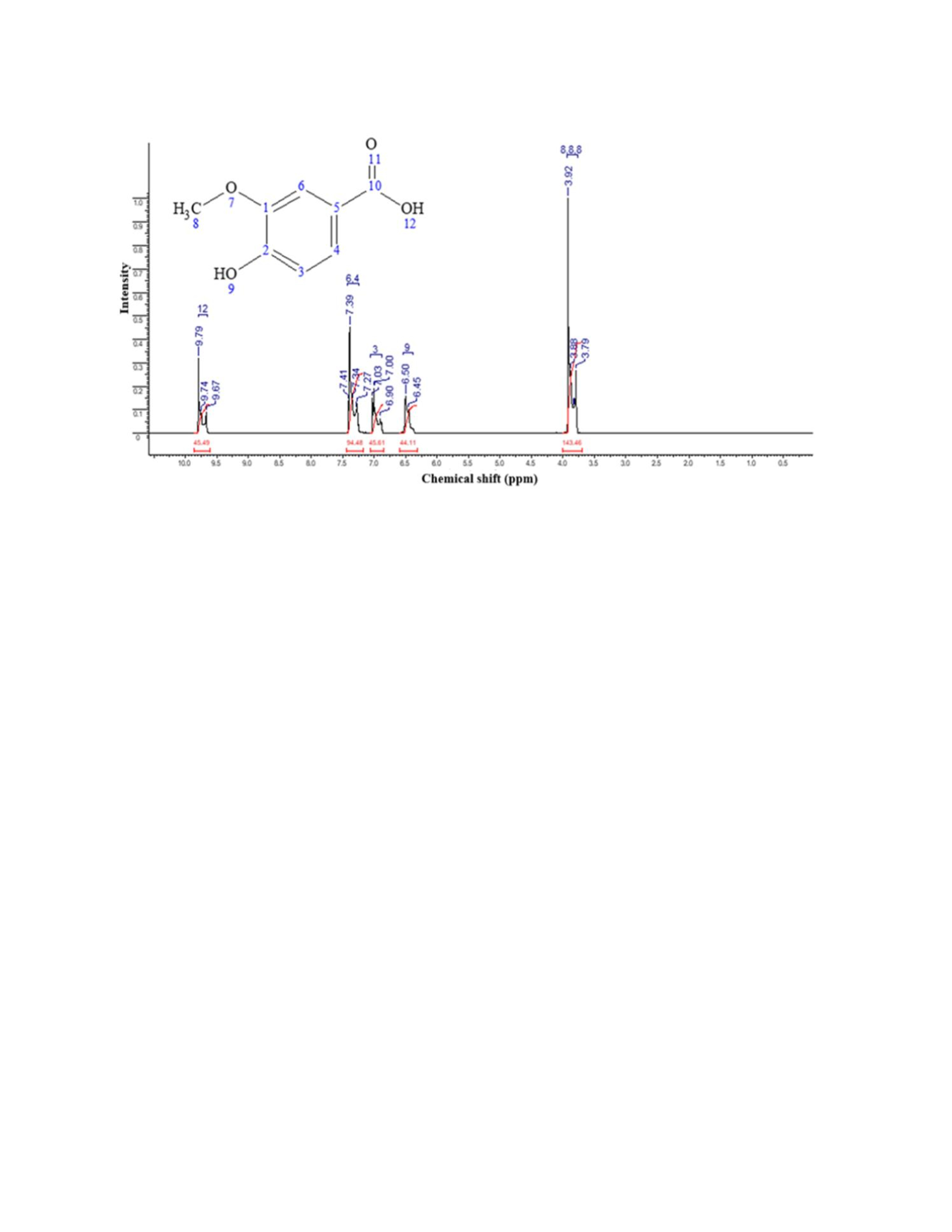

Supplement: Supplementary file 1 [file ijms-26-10301-s001.zip › Figure S2. 1H NMR spectrum of compound 2.1 (vanillic acid) recorded in CDCl3 (characteristic proton signals for aromatic hydrogens (H-3, H-4, H-6), methoxy gro.jpg]

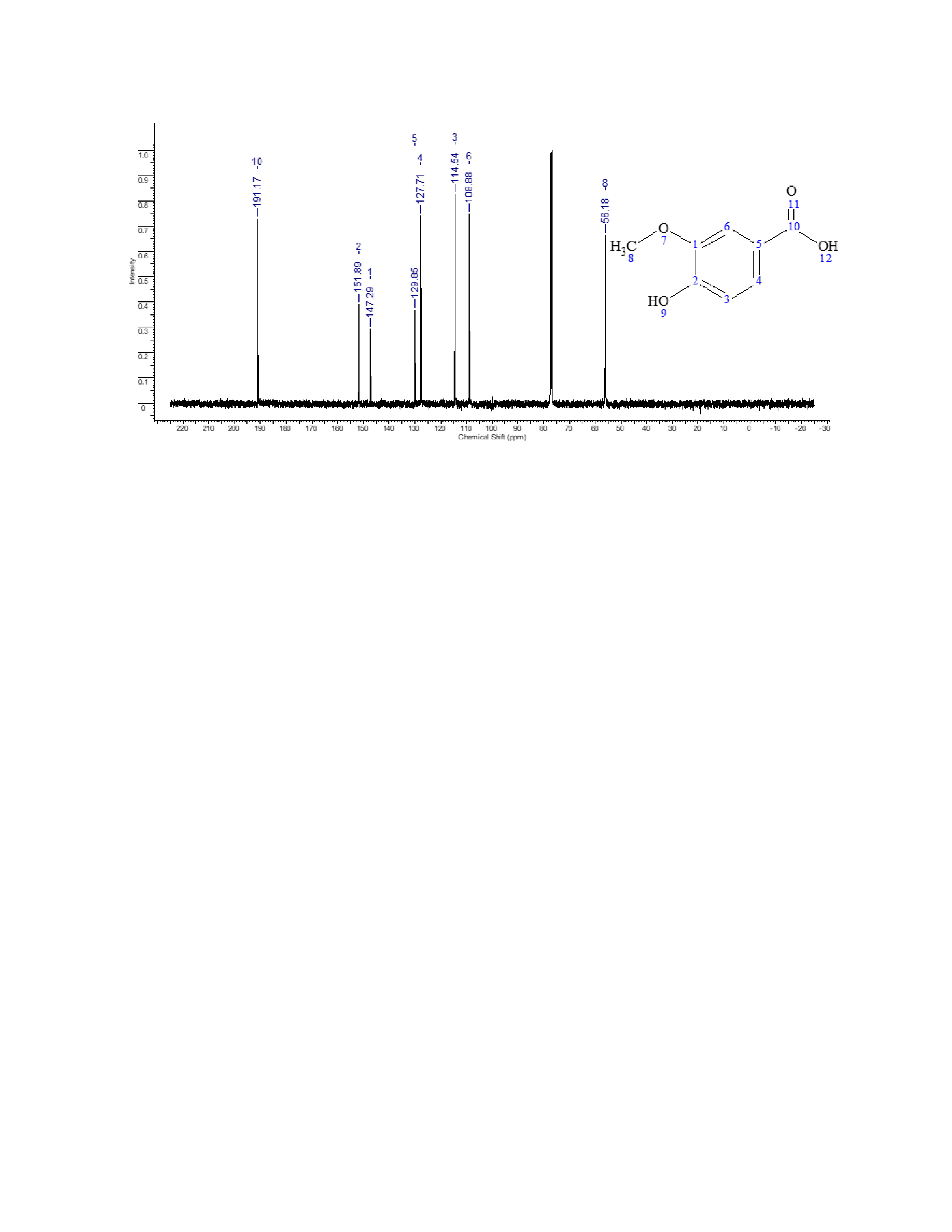

Supplement: Supplementary file 1 [file ijms-26-10301-s001.zip › Figure S3. 13C NMR spectrum of compound 2.1 in CD3OD, showing characteristic carbon resonances corresponding to aromatic carbons, carboxylic group, hydroxylated.jpg]

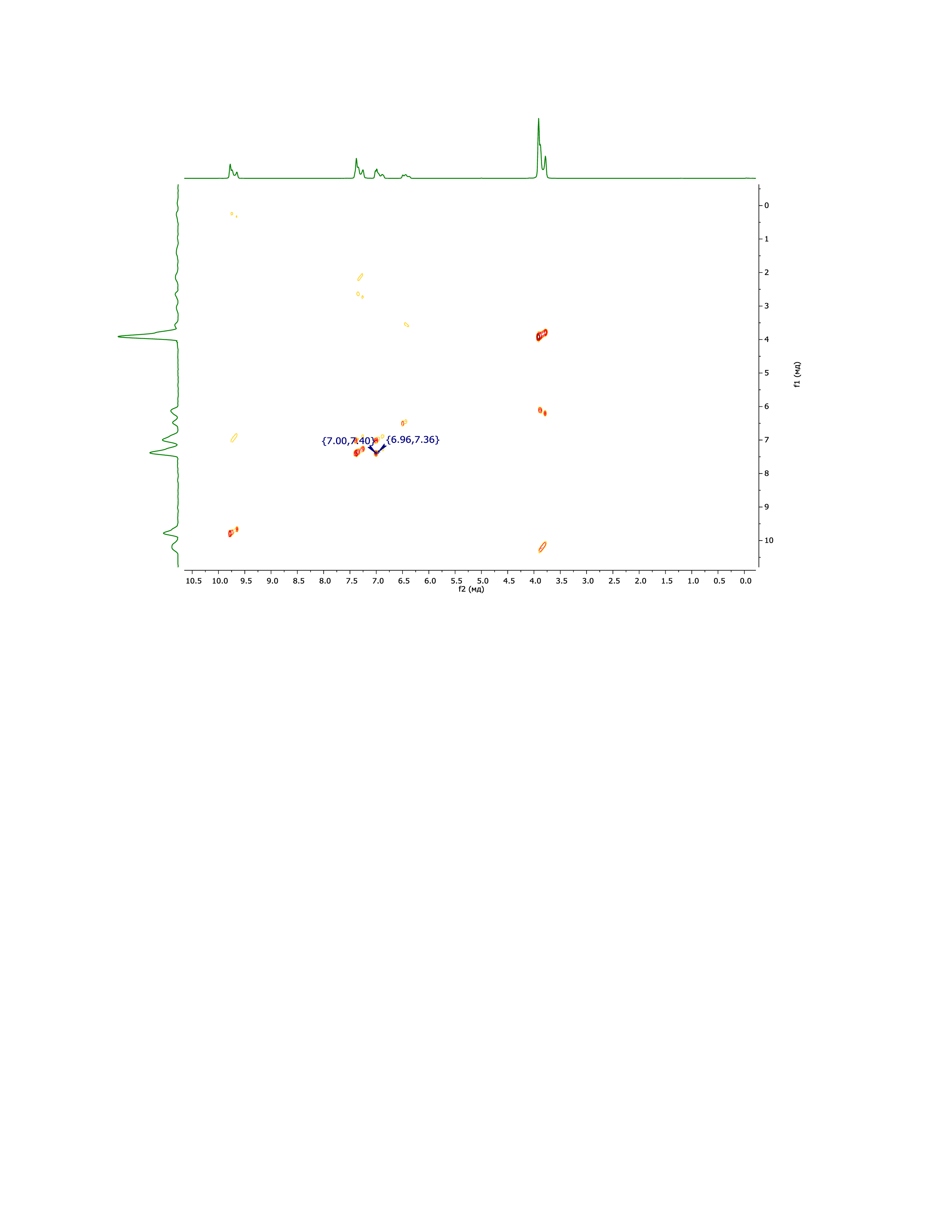

Supplement: Supplementary file 1 [file ijms-26-10301-s001.zip › Figure S4. 2D COSY spectrum of compound 2.1 (vanillic acid) showing scalar (J) couplings between aromatic protons and confirmation of the spin system within the.jpg]

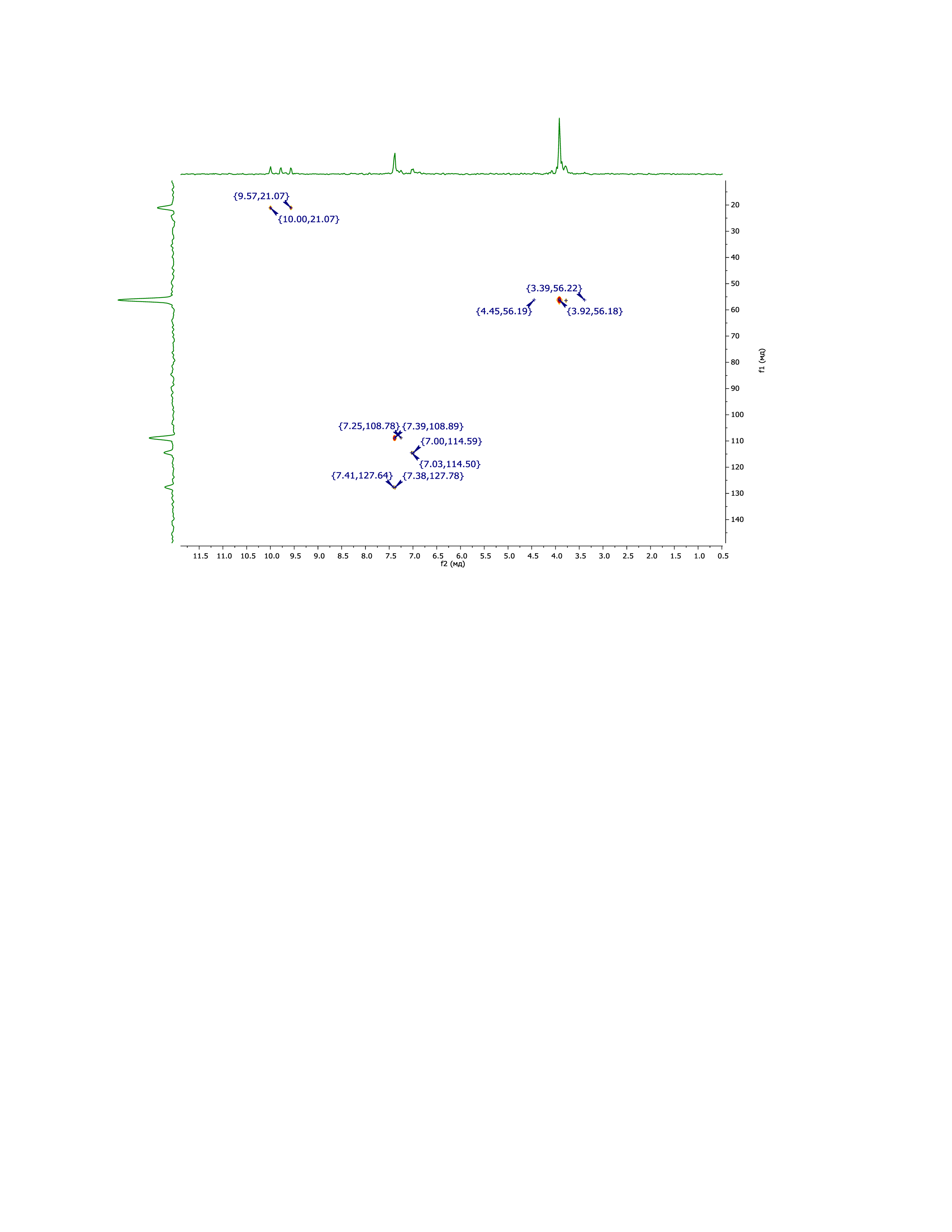

Supplement: Supplementary file 1 [file ijms-26-10301-s001.zip › Figure S5. 2D HSQC spectrum of compound 2.1 (vanillic acid) displaying direct one-bond correlations between proton and carbon nuclei, confirming assignments of.jpg]

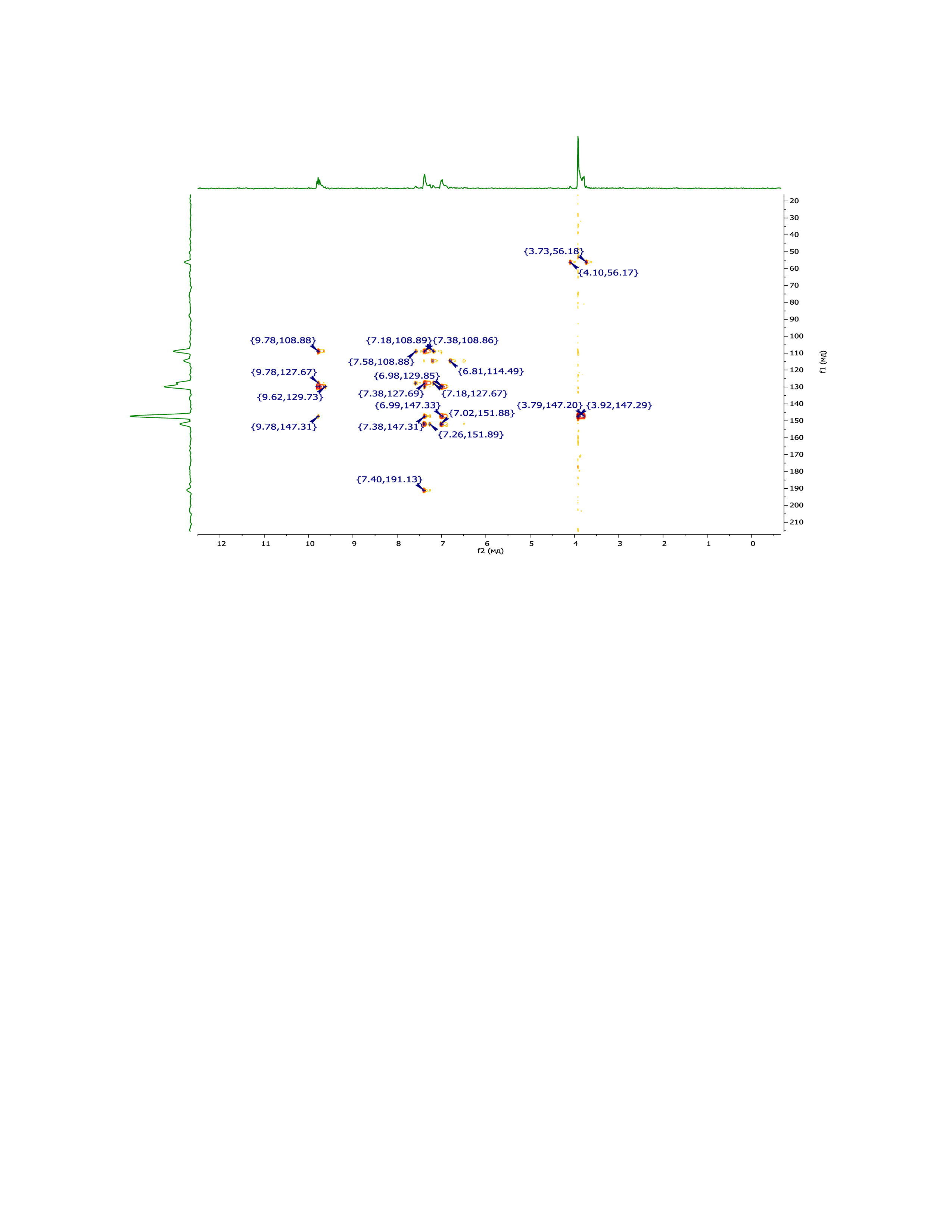

Supplement: Supplementary file 1 [file ijms-26-10301-s001.zip › Figure S6. 2D HMBC spectrum of compound 2.1 (vanillic acid) showing long-range 1H–13C heteronuclear correlations, supporting structural connectivity among aroma.jpg]

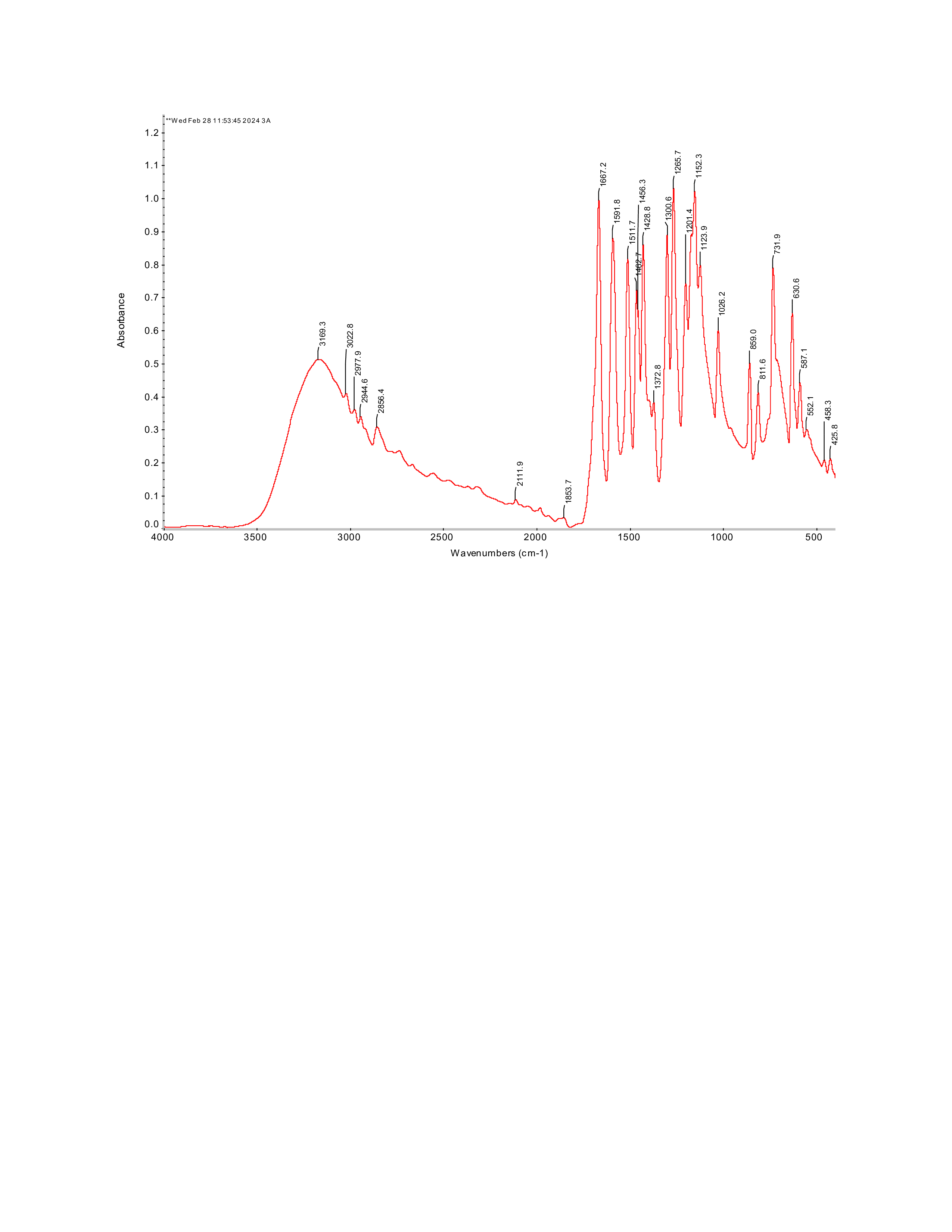

Supplement: Supplementary file 1 [file ijms-26-10301-s001.zip › Figure S7. IR spectrum of compound 2.1..jpg]

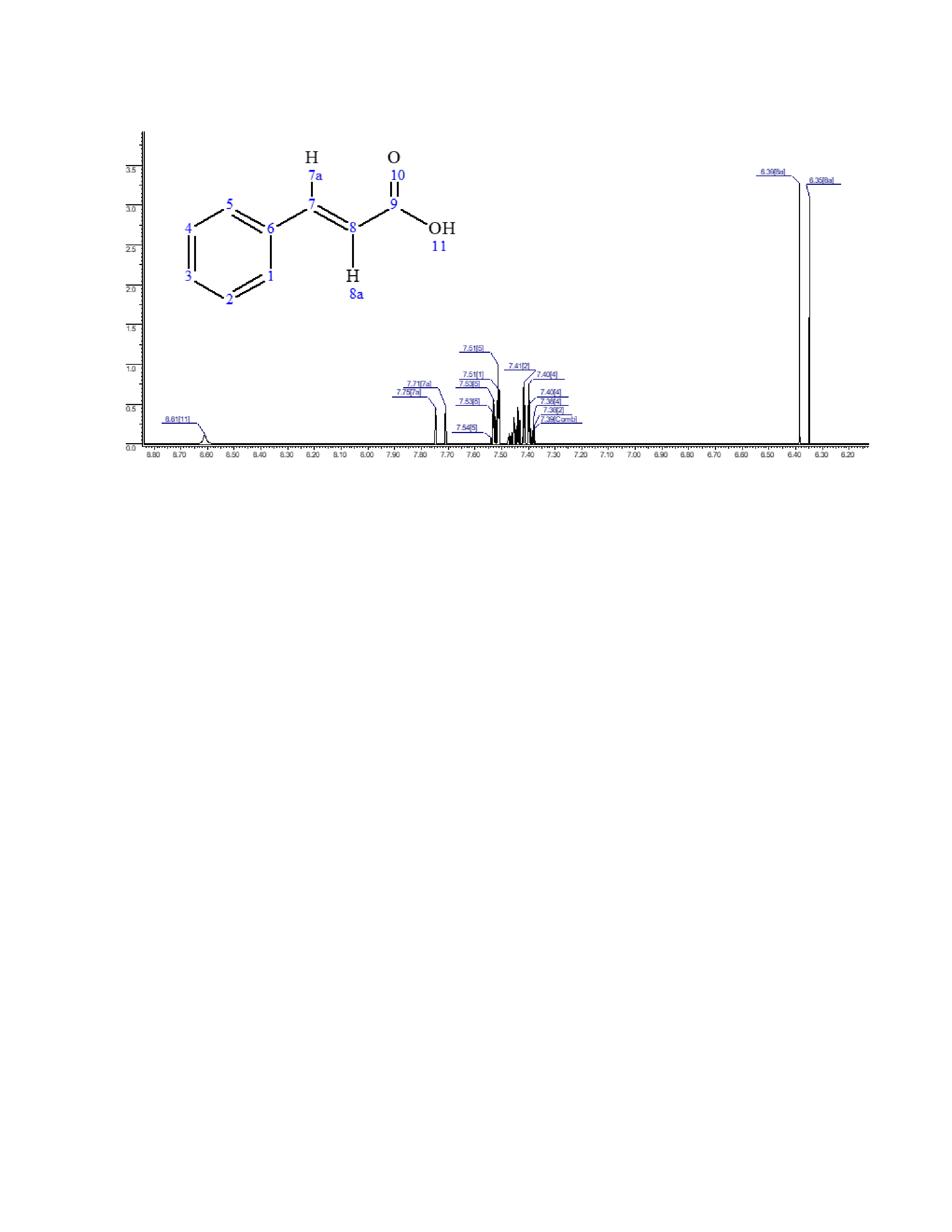

Supplement: Supplementary file 1 [file ijms-26-10301-s001.zip › Figure S8. 1H NMR spectrum of compound 2.2 (cinnamic acid) recorded in CD3OD..jpg]

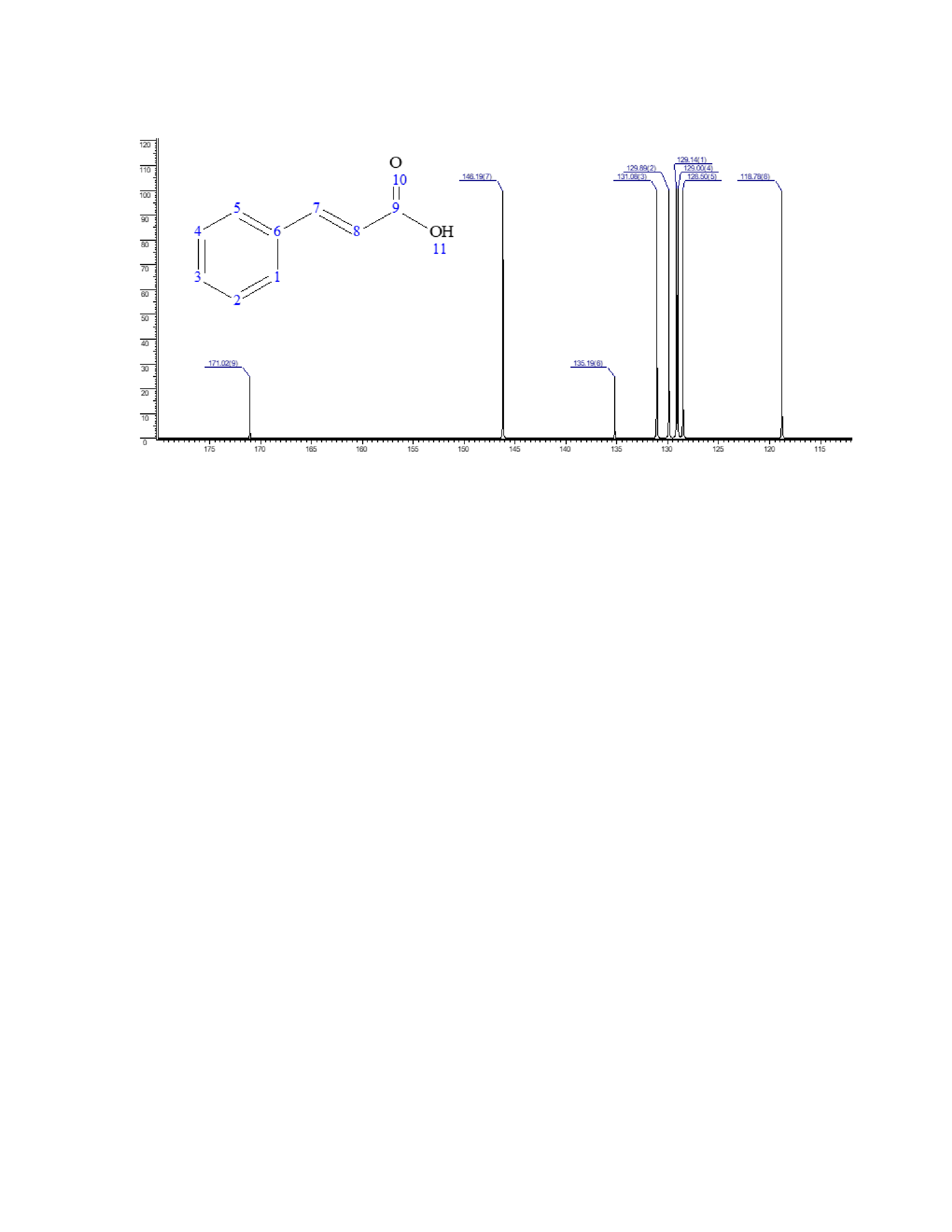

Supplement: Supplementary file 1 [file ijms-26-10301-s001.zip › Figure S9. 13C NMR spectrum of compound 2.2 (cinnamic acid) recorded in CD3OD..jpg]
